# Neuromuscular junction failure in sarcopenia is linked to NaV1.4 loss and reversed by ClC-1 inhibition

**DOI:** 10.1172/JCI190646

**Published:** 2026-07-09

**Authors:** W. David Arnold, Jeanette Jeppesen Morgen, Pernille Bogetofte Thomasen, Martin Broch-Lips, Leatha A. Clark, Thomas Groennebaek, Martin Skov, Jeppe Blichfeldt Winther, Abdullah Ramadan, Philippa A. Rust, Jessica H. Myers, Fereshteh B. Darvishi, Anna R. Dashtmian, Lauren A. Fish, Deepti Chugh, Jane Bold, Jorge Quiroz, John Hutchison, Hiroshi Nishimune, Ross A. Jones, Xueyong Wang, Justin R. Fallon, Thomas H. Gillingwater, Mark M. Rich, Thomas Holm Pedersen, Brian C. Clark

**Affiliations:** 1 NextGen Precision Health and; 2 Department of Physical Medicine and Rehabilitation, University of Missouri, Columbia, Missouri, USA.; 3 NMD Pharma A/S, Aarhus, Denmark.; 4 Ohio Musculoskeletal and Neurological Institute and; 5 Department of Biomedical Sciences, Ohio University Heritage College of Osteopathic Medicine, Athens, Ohio, USA.; 6 Edinburgh Medical School, Biomedical Sciences, College of Medicine and Veterinary Medicine, University of Edinburgh, Edinburgh, United Kingdom.; 7 College of Sciences and Health Professions, King Saud bin Abdulaziz for Health Sciences, Jeddah, Saudi Arabia. .; 8 Department of Neuroscience, Cell Biology and Physiology, Wright State University, Dayton, Ohio, USA.; 9 Department of Neuroscience and Carney Institute for Brain Science, Brown University, Providence, Rhode Island, USA.; 10 Indian Institute of Technology, Kanpur, India; 11 Department of Biomedicine, Aarhus University, Aarhus, Denmark.

**Keywords:** Aging, Neuroscience, Excitation contraction coupling, Sodium channels, Synapses

## Abstract

Sarcopenia is the age-related loss of muscle strength and size that leads to mobility limitations and loss of independence in older adults. The underlying cellular mechanisms remain unclear, and treatments are limited. As the critical interface between the nervous system and muscle, the neuromuscular junction (NMJ) is essential for muscle activation and force production. Here, we demonstrate that weak older individuals exhibited NMJ transmission failure that correlated with muscle weakness severity. Preclinical experiments showed similar NMJ transmission failure in aged rodents that was associated with localized loss of muscle fiber excitability at the NMJ. This excitability defect, distinct from potential synaptic cholinergic transmission abnormalities, represents a disease mechanism of sarcopenia. Across species, immunohistochemistry identified a localized reduction in the voltage-gated sodium channel specific for skeletal muscle (NaV1.4) at the postsynaptic NMJ membrane. Acute NaV1.4 inhibition with μ-conotoxin GIIIB in adult rats reproduced findings of NMJ transmission failure observed in aged rodents and humans. Finally, ClC-1 chloride ion channel inhibition enhanced muscle excitability and improved NMJ transmission and muscle function in old rodents. Together, these findings demonstrate that NMJ transmission deficits are a key, reversible driver of sarcopenia and reveal a therapeutic target for addressing muscle weakness in aging.

## Abstract 2

Sarcopenia is linked to neuromuscular junction (NMJ) transmission failure. This study identifies NMJ deficits and a potential therapeutic strategy to improve muscle function in aging.

## Introduction

Despite increased lifespan over the past century, healthy life expectancy (healthspan) has not kept pace (1, 2), with mobility limitations contributing to the gap between life expectancy and healthspan. Sarcopenia refers to age-related loss of muscle strength and size that plays a major role in declining mobility, leading to a higher fall risk, reduced quality of life, and increased healthcare expenditure in older adults (3–11). Recent consensus highlights that muscle weakness, rather than muscle mass loss, is the primary feature of sarcopenia (7, 12). Although the mechanisms behind age-related muscle weakness remain unresolved, emerging evidence suggests that dysfunction at the neuromuscular junction (NMJ), the critical link between the nervous system and muscle, could be a key contributor (13). NMJ transmission failure, which compromises the ability of motor neurons to activate muscle contraction, is known to contribute to weakness in several diseases (14–16). Consequently, understanding NMJ deficits in aging has become a focal point for sarcopenia research (17–19).

Evidence of NMJ failure in aging primarily comes from rodent studies (19–21). For instance, aged rats show reduced force generation during peripheral nerve stimulation compared with direct muscle stimulation, indicating NMJ transmission deficits (22). Recent work using single-fiber electromyography (SFEMG) further supports these findings by showing NMJ transmission failure in aged rodents (20, 21). However, direct evidence of NMJ dysfunction from older humans remains scarce, as most studies use indirect methods to assess NMJ function, yielding inconsistent results (23–27). To date, no studies have directly examined NMJ transmission failure in older adults using SFEMG.

NMJ transmission relies on a series of steps triggered by a neuronal action potential (AP) that releases acetylcholine, leading to endplate potential (EP) generation (28). Normally, the EP exceeds the voltage threshold for activation of voltage-gated sodium channels (NaV1.4), which are selective for muscle fibers and highly expressed at the NMJ. Once activated, the inward current through NaV1.4 channels is responsible for the rapid depolarization and propagation of the muscle fiber AP. In healthy muscle, the amplitude of the EP consistently exceeds the threshold for NaV1.4 activation, ensuring reliable muscle contraction (28). This reliability is due not only to the high EP amplitude but also to the greater excitability of muscle fibers at the NMJ compared with their excitability in regions outside the NMJ (extrajunctional regions) (29). This elevated muscle excitability at the NMJ, compared with the extrajunctional muscle fiber membrane, reflects higher expression of NaV1.4 channels at the NMJ and will be referred to here as “gain” (29, 30). Interestingly, while aging rodent studies show preserved or even heightened EP amplitudes, suggesting an increased safety factor, NMJ transmission failure is still observed in preclinical SFEMG studies (31–34). This apparent contradiction warrants further investigation into the underlying mechanisms of transmission failure in aging and the role that muscle excitability plays in this process.

The first goal of this study was to investigate NMJ transmission dysfunction in weak older adults (20, 21) by using SFEMG to provide direct evidence for NMJ transmission failure in a clinically relevant muscle. We also sought to determine whether any NMJ transmission dysfunction was associated with impaired muscle strength (normalized to muscle mass) (35). Second, to explore the underlying mechanisms behind NMJ dysfunction in aging, we identified a loss of localized muscle fiber excitability (reduced gain) at the NMJ in old rodents compared with adult rodents, which was associated with reduced NaV1.4 channel protein level at the motor endplate. This phenomenon of reduced NaV1.4 expression level at the NMJ was also observed in aged humans. To directly determine whether reduced NaV1.4 function could contribute to NMJ transmission failure, we acutely inhibited NaV1.4 channels in adult rats using μ-conotoxin GIIIB. These experiments in adult rats revealed SFEMG abnormalities of increased jitter and blocking, akin to those observed in aged rodents and humans. Finally, we investigated a therapeutic approach aimed at increasing postsynaptic excitability by inhibiting the ClC-1 ion channel, a channel involved in stabilizing muscle membrane excitability, which enhanced muscle function in aged rodents. Together, these findings suggest that NMJ transmission deficits are a key contributor to neuromuscular dysfunction in sarcopenia and highlight ClC-1 channels as a therapeutic target for addressing muscle weakness in aging.

## Results

### Clinical assessment of NMJ transmission failure

#### Older adults with clinically meaningful weakness exhibit notable impairments in NMJ transmission.

We first investigated whether older humans with clinically meaningful weakness indicative of sarcopenia exhibited impairments in NMJ transmission. Ten older adults who self-reported physical limitations were recruited (mean age: 85.9 ± 4.4 years; range: 83–96 years; 40% female). A control group of 8 young and middle-aged adults was tested for comparison (mean age: 29.5 ± 8.7 years; range: 20–45 years; 38% female). Descriptive statistics of study participants are detailed in Table 1 (inclusion and exclusion criteria are in Supplemental Table 1; supplemental material available online with this article; https://doi.org/10.1172/JCI190646DS1). To assess muscle strength, we measured participants’ isokinetic leg extensor strength at 60°/sec. We also normalized this strength measure to their quadriceps fat-subtracted muscle volume on MRI (35). To further characterize neuromuscular health in study participants, we also quantified handgrip strength, overall physical function, and body composition using dual-energy x-ray absorptiometry scans to quantify lean mass (Table 1).

**Figure 1:**
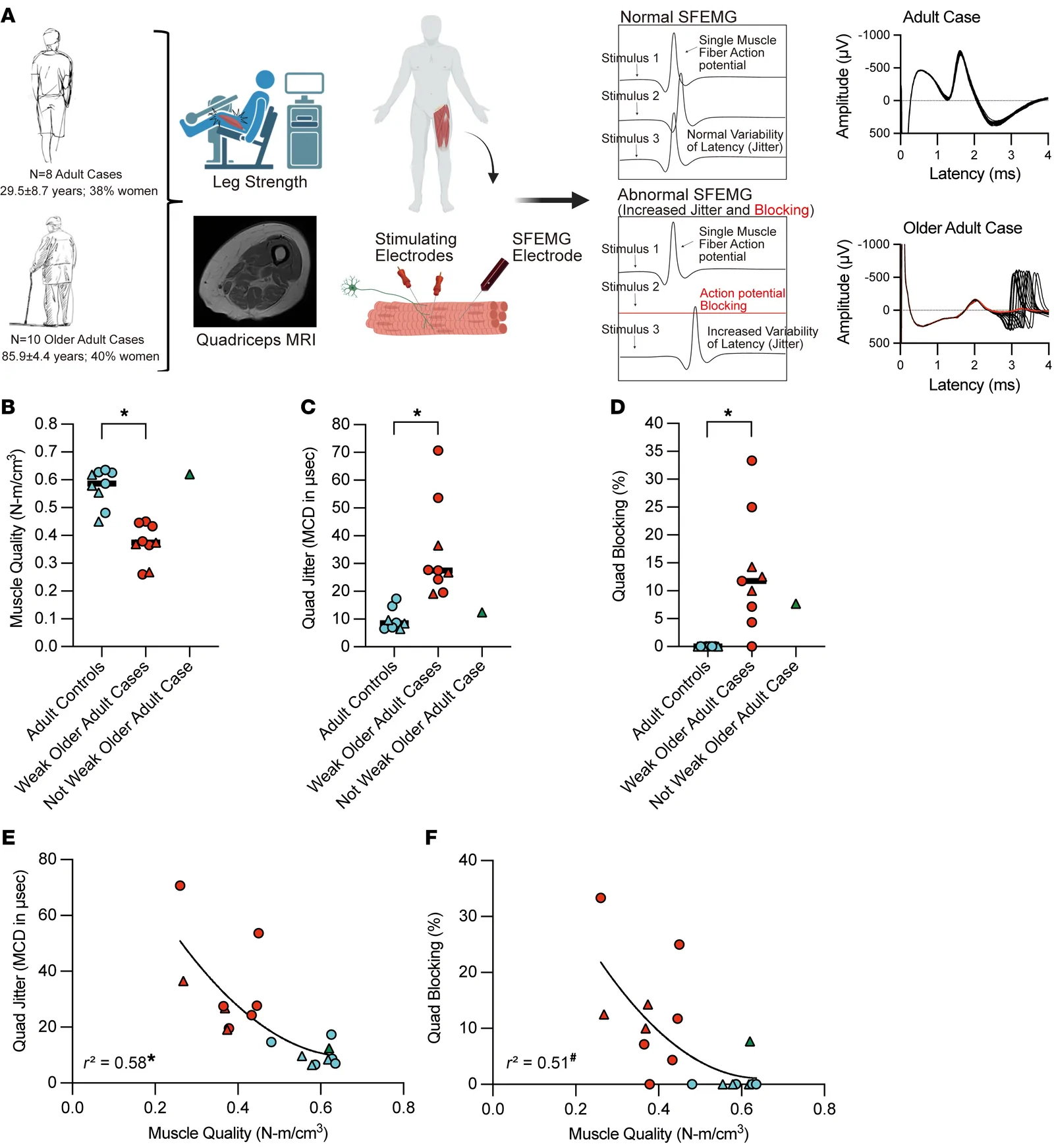
Older adults with clinically meaningful muscle weakness exhibit notable impairments in NMJ transmission. (**A**) Overview of key muscle and electrophysiological outcomes. Images on the far right represent example SFEMG traces from an adult (top) and a weak older adult (bottom). The arrows denote the negative peaks of the superimposed single fiber APs, and the red traces represent blocked APs. Note: The absolute latency between stimulation and single fiber AP generation is determined by the relative distance between the stimulating and recording electrodes. (**B**) Levels of isokinetic leg extensor muscle strength normalized to MRI-derived values of quadriceps muscle volume (fat subtracted) — referred to as Muscle Quality — were 44% lower in weak older adult cases compared with healthy adults acting as controls. *P < 0.001. (**C**) Jitter — the variability in the arrival time of APs to the recording electrode between consecutive electrical discharges — was greater in weak older adults when compared with that in adults acting as controls. *P = 0.003. (**D**) Neuromuscular junction blocking (indicated as the percentage of synapses with transmission failure) was prominently observed in weak older adults. *P = 0.005. (**E** and **F**) The degree of jitter (*P < 0.002) and blocking (^#^*P* = 0.007) were both associated with impaired muscle strength (normalized to muscle volume). *P* values reported in **B**–**D** are derived from independent sample *t* tests with Welch’s correction for equal variances not assumed, and R^2^ values reported in **E** and **F** are derived from quadratic regression. Solid lines in **B**–**D** represent the mean values. Triangles indicate female participants; circles indicate male participants.

Stimulated SFEMG was used to assess NMJ transmission. During SFEMG, a pair of monopolar needle electrodes were inserted in the motor point region of the vastus lateralis to allow stimulation of terminal nerve twigs of the femoral nerve. A highly selective SFEMG recording needle electrode was inserted into the vastus lateralis approximately 2 cm distal to the stimulation electrodes. Nerve stimulation was delivered at 10 Hz, and single muscle fiber AP responses (all-or-none responses ≥200 μV amplitude and < 300 μs rise times) were recorded following 50–100 consecutive stimulations of each synapse (Figure 1A). SFEMG parameters of jitter, which is the variability in the arrival time of APs to the recording electrode between consecutive electrical discharges, and intermittent blocking (i.e., complete NMJ transmission failure) were quantified (Figure 1A). One of the older adults did not exhibit clinically meaningful leg extensor weakness, whereas 9 were classified as having clinically meaningful leg extensor weakness based on previously published cut points. Of these, 7 were classified as a high-risk and 2 as moderate-risk for subsequently developing severe mobility limitations defined as two consecutive reports of a lot of difficulty or inability to walk one-quarter of a mile or climb 10 steps) (36). In addition, 7 older participants were also classified as having handgrip weakness based on the most recent European consensus definition for sarcopenia (7). The older participants also exhibited low levels of physical performance, as assessed by the short physical performance battery (e.g., 50% scored ≤ 8) (Table 1). Descriptive characteristics of the participants’ leg extensor muscle strength (normalized to quadriceps femoris muscle volume) are provided in Figure 1B. Consistent with prior rodent findings (20, 21), we observed that, on average, older adults with leg extensor weakness (*n* = 9) exhibited approximately 250% higher mean jitter values than the individuals acting as controls (Figure 1C). Moreover, most of the older adults exhibited prominent levels of blocking (Figure 1D). The degree of jitter and blocking were both inversely associated with volitional muscle strength (normalized to muscle volume) (Figure 1, E and F).

### Mechanistic investigation of age-related NMJ transmission failure

#### Age-related loss of postsynaptic excitability at the NMJ despite maintained whole muscle fiber excitability.

To address the discrepancy between the unchanged or enhanced EP amplitude reported in older animals (20, 32), which imply an increased safety factor, and indications of NMJ transmission failure on SFEMG recordings, we conducted a series of experiments to assess the excitability of isolated single muscle fibers using intracellular microelectrodes in both adult and old C57BL/6 mice and found no discernible differences. Here, extensor digitorum longus (EDL) muscle fiber excitability was studied in 10 adult mice (6–7 months, 5 males, 5 females) and 9 old mice (26–27 months, 5 males, 4 females). No sex differences were observed, so data were pooled. No differences in resting potential, passive membrane properties, or AP parameters were observed between adult and old mice (Table 2). In a subset of old mice, we measured the ability of muscle fibers to sustain AP firing throughout a 2-second train of direct muscle stimulation delivered at 40 Hz. In all of the 7 old mice studied, all fibers were able to fire APs in response to all 80 stimuli delivered, demonstrating that there is no defect in the ability of whole fibers from old mice to fire APs repetitively following direct muscle stimulation.

**Figure 2:**
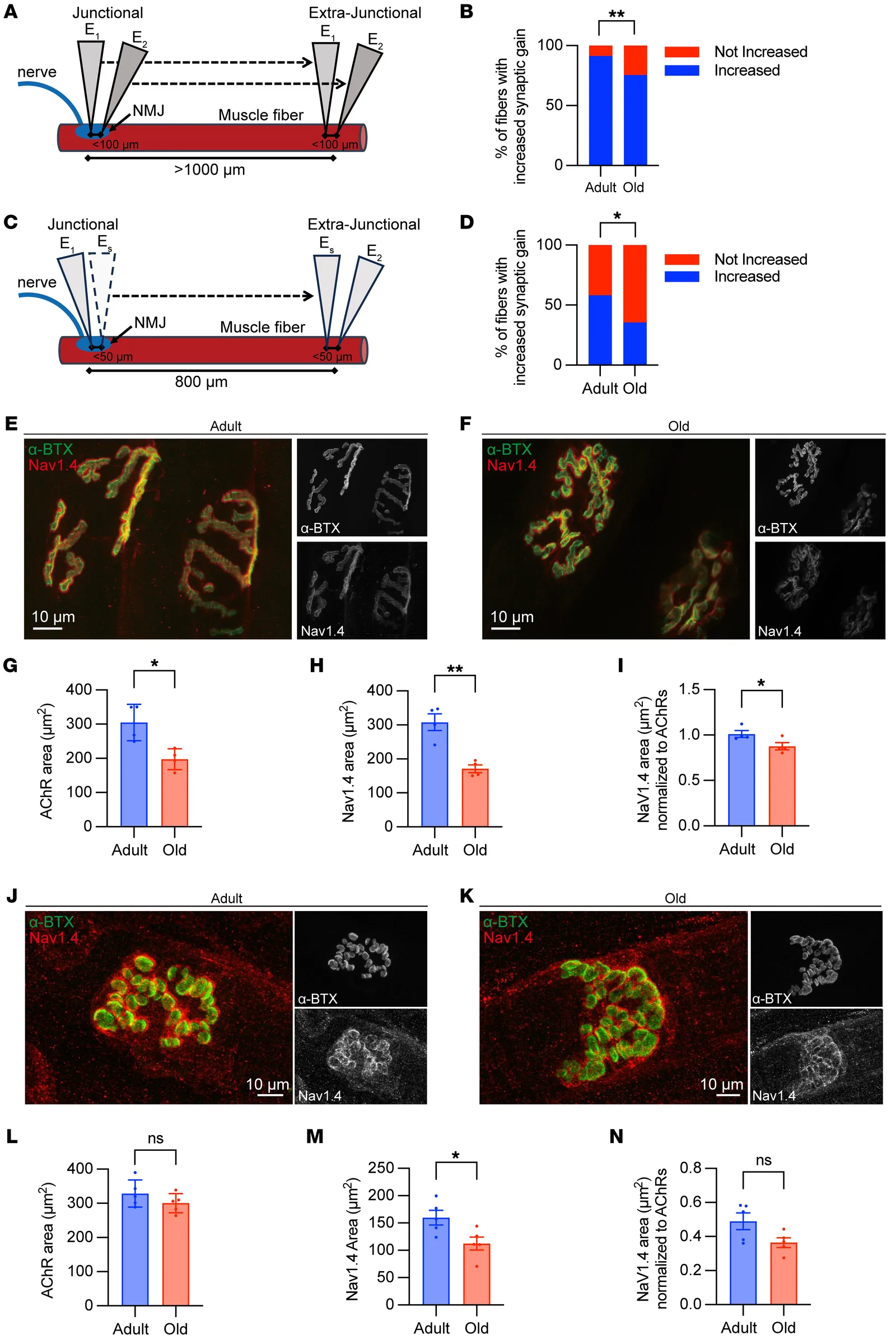
Age-related decline in localized postsynaptic excitability and reduced NaV1.4 channel distribution at the NMJ. (**A** and **B**) Excitability was assessed in adult and old mice using a 2-electrode approach in which electrodes E1 and E2 were positioned together near the NMJ (junctional, within 1,000 μm) and then repositioned farther from the NMJ (extrajunctional, >1,000 μm). Older mice exhibited significantly fewer fibers with localized enhanced excitability at the NMJ (gain) compared with adult mice. Gain was defined by a higher stimulation requirement in extrajunctional regions than at the NMJ to trigger an action potential (AP). Total fibers measured: adult, 81; old, 69. (**C** and **D**) Similar reductions in excitability gain were observed in old rats (23–24 months, *n* = 7) compared with adult rats (6–7 months, *n* = 5) using a 3-electrode approach. The centralstimulating electrode (E_s_) was positioned near the NMJ (junctional within 50 μm) and then farther from the NMJ (extrajunctional >800 μm), and APs were elicited by current injection through E_s_. Total fibers measured: adult, 36; old, 45. (**E** and **F**) Representative confocal images of sternomastoid NMJs from adult (3–4 months, *n* = 4) and old (26–28 months, *n* = 4) mice. AChRs were labeled with α-bungarotoxin (green) and NaV1.4 with specific antibodies (red). (**G**–**I**) Quantitative analysis in mouse NMJs: (**G**) AChR area, (**H**) NaV1.4 area, and (**I**) NaV1.4 area normalized to AChR area. Compared with adults, aged mice showed significant reductions across all measures. (**J** and **K**) Representative confocal images of lumbrical NMJs from adult (5.5–7 months, *n* = 5) and old (21–23 months, *n* = 5) rats. (**L**–**N**) Quantitative analysis in rat NMJs: (**L**) AChR area, (**M**) NaV1.4 area, and (**N**) NaV1.4 area normalized to AChR area. Old rats showed significant reductions in NaV1.4 area from adult rats. (**G**–**I** and **L**–**N**) Each data point represents mean values per animal from 25–27 NMJs per mouse and 23–25 NMJs per rat. Data are shown as mean ± SEM. *P < 0.05, **P < 0.01 using unpaired *t* tests. Scale bars: 10 μm.

After confirming no loss of excitability, at the whole-fiber level, in isolated muscle fibers, we next investigated alterations of excitability localized at the NMJ (Figure 2). Using intracellular electrodes, we triggered and recorded APs both near the endplate (junctional) and away from the endplate (extrajunctional) in both adult and old male C57BL/6 mice and Wistar rats (Figure 2). Previous studies have shown that the excitability of healthy muscle fibers is highest around the NMJ due to a larger current through NaV1.4 at the junction compared with extrajunctional regions (30). Junctional enhancement (gain) was indicated by the observation that a smaller stimulus was required to initiate an AP at the NMJ compared with regions away from the NMJ (localized excitability enhancement or “gain”). Such gain was more frequently observed in fibers from adult mice as compared with muscle fibers in older mice (Figure 2, A and B). We also extended these investigations to adult and old rats using a similar recording approach. Consistent with the findings in mice, older rats also demonstrated significantly reduced gain at NMJ (Figure 2, C and D). To address whether these age-related differences in endplate excitability could be related to loss of resting membrane potential during repeated electrode impalements, we compared the correlation between synaptic gain in individual fibers and the degree of depolarization across repeated impalements of muscle fibers (Supplemental Figure 1). These analyses showed no meaningful correlation between resting membrane potential and synaptic gain validating that the decreased gain in old muscle fibers could not be ascribed to an increased NaV1.4 inactivation caused by more pronounced depolarization induced by repeated electrode impalement. Collectively, low-gain fibers occurred predominantly in old mice, supporting that reduction of NaV1.4 current at NMJ could introduce age-related synaptic deficit (37).

#### Age-dependent reduction of NaV1.4 enrichment at the NMJ.

Voltage-gated sodium channel NaV1.4 is highly enriched at the postsynaptic membrane of the NMJ, where its clustering at the endplate increases local membrane excitability and secures reliable AP initiation in response to EPs (30). Prior studies have demonstrated that NaV1.4 concentration within the perijunctional folds contributes critically to the safety factor of neuromuscular transmission (30). We therefore examined whether age-related alterations in NaV1.4 channel density at the NMJ could contribute to functional transmission defects observed with aging.

To quantify NaV1.4 distribution at the NMJ, immunohistochemical staining and confocal microscopy morphometric analysis were performed in muscles from adult and aged rodents. We measured acetylcholine receptor (AChR) area, NaV1.4^+^ area, and NaV1.4 area normalized to AChR area in sternomastoid muscles from mice and lumbrical muscles from rats. In aged mice, all 3 parameters were significantly reduced compared with those in adult controls (Figure 2, E–I), indicating both reduced NMJ size and diminished NaV1.4 enrichment. In aged rats, AChR area was preserved, whereas NaV1.4 area was significantly reduced. NaV1.4 area normalized to AChR area showed a similar downward trend but did not reach statistical significance (Figure 2, J–N). These findings suggest that aging preferentially affects NaV1.4 localization at the NMJ, with species- and muscle-dependent differences.

To determine whether these changes reflect fiber-type–specific regulation or redistribution along the myofiber, we analyzed longitudinal cryosections of fast-twitch EDL and slow-twitch soleus muscles from adult and aged mice. NaV1.4 expression was quantified at junctional and extrajunctional regions and normalized to AChR-defined endplate area. In the predominantly fast-twitch EDL, NaV1.4 enrichment at the NMJ was significantly reduced in aged mice, while extrajunctional NaV1.4 levels were unchanged (Supplemental Figure 2, A–D), indicating a selective junctional defect rather than global channel loss. In contrast, NaV1.4 distribution in the slow-twitch soleus was preserved with aging, with no evidence of redistribution or junctional depletion (Supplemental Figure 2, C–M). Thus, aging appears to alter NaV1.4 localization in a muscle-dependent manner, preferentially affecting fast-twitch muscle. To determine whether reduced junctional NaV1.4 reflects transcriptional downregulation, we quantified *Scn4a* transcript levels in EDL and soleus muscles using digital PCR (Supplemental Figure 3). Whole-muscle *Scn4a* mRNA levels were unchanged with age in EDL but showed a modest yet significant reduction in soleus. The absence of mRNA decline in EDL despite reduced junctional NaV1.4 protein supports a post-transcriptional or trafficking-based mechanism in fast-twitch muscle, whereas soleus demonstrates modest transcriptional regulation without detectable junctional protein loss. Together, these data indicate muscle-specific regulation of NaV1.4 with aging rather than uniform channel depletion.

#### NaV1.4 loss at the human NMJ occurs despite preserved morphology.

**Figure 3:**
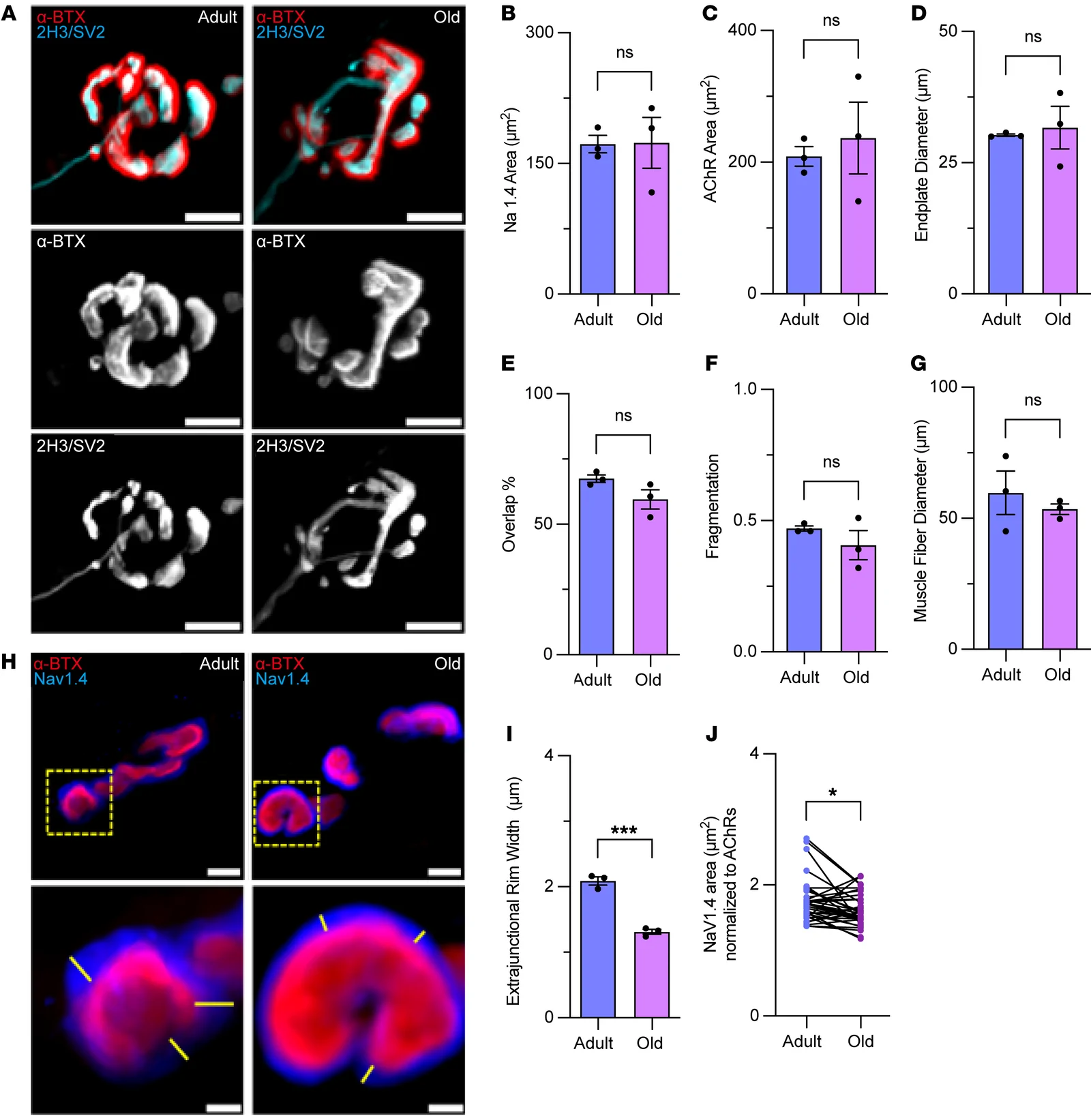
Absence of anatomical denervation but presence of junctional and parajunctional NaV1.4 disruption in human NMJs with aging. (**A**) Representative confocal images of NMJs from adult and old human dorsal interosseous muscles. AChRs were labeled with α-bungarotoxin (red), while neurofilament and synaptic vesicles were labeled with 2H3 and SV2 (cyan). Individual channels are shown in gray scale below. These representative NMJ images are also shown in Supplemental Figure 4A. Scale bars: 10 μm. (**B**–**G**) Morphology quantification in adult (blue) versus old (violet) human samples. (**B**) Nerve terminal area. (**C**) AChR area. (**D**) Endplate diameter. (**E**) Nerve-endplate overlap. (**F**) Postsynaptic fragmentation. (**G**) Muscle fiber diameter. In **B**–**G**, each data point represents the mean of ≈40 NMJs per individual (*n* = 3 per group); no age-related differences were detected. (**H**) Representative confocal images of NMJs from adult (left) and old (right) human dorsal interosseous muscles (same biopsies as in **A**). AChRs were labeled with α-bungarotoxin (red), and NaV1.4 is shown in blue. At adult NMJs, the NaV1.4 signal extends beyond the AChR-defined postsynaptic region, forming a parajunctional rim (denoted by yellow dashed lines). By contrast, in aged NMJs this NaV1.4 rim was markedly reduced, with NaV1.4 confined to the AChR territory (indicated by yellow bars). Scale bars: 10 μm (top); 2.5 μm (bottom). (**I**) Parajunctional NaV1.4 rim width (≈18 NMJs per individual, *n* = 3 per group) revealed a significant reduction in older individuals. (**J**) NaV1.4 signal area normalized to AChR area after matching adult and old NMJs. Each old NMJ was matched to the adult NMJ with the most comparable AChR area, using a nearest-neighbor algorithm; pairs differing by >10% were excluded. Lines connect matched NMJs from different individuals. Data are shown as mean ± SEM. Unpaired *t* tests or Mann-Whitney *U* tests were used for **B**–**G** and **I**, and paired *t* test were used for **J**. *P < 0.05, ***P < 0.001.

To assess translational relevance, we examined NaV1.4 distribution in surgical biopsy samples from dorsal interosseous muscles obtained during reconstructive procedures (Supplemental Table 2). Consistent with prior analyses of lower-limb muscles in aging, detailed morphometric assessment revealed no overt structural denervation or gross disruption of NMJ architecture in older adults (Figure 3, A–G, and Supplemental Figure 4) (38).

Despite preserved structural integrity, NaV1.4 protein localization at the NMJ was significantly reduced in older individuals. Quantification of the parajunctional NaV1.4 rim — reflecting channel enrichment within secondary postsynaptic folds extending beyond AChR-defined endplate boundaries — demonstrated a significant reduction in rim width in older samples (Figure 3, H and I). In a subset analysis (~18 NMJs per individual), total NaV1.4 area was similarly reduced. To control for potential confounding by NMJ size differences, we implemented a one-to-one nearest-neighbor matching strategy, pairing each older NMJ with a size-matched younger NMJ (AChR-defined area difference, <10%). Even after rigorous normalization, NaV1.4 area relative to AChR area remained significantly decreased in older adults (Figure 3J). These findings demonstrate selective loss of junctional NaV1.4 with aging in humans, independent of gross synaptic remodeling. Collectively, the rodent and human data indicate that aging is associated with reduced postsynaptic NaV1.4 enrichment at the NMJ despite preserved overall morphology. This supports a model in which age-related neuromuscular transmission failure reflects impaired postsynaptic excitability, consistent with increased jitter and impulse block observed clinically in older adults.

#### Acute NaV1.4 inhibition recapitulates age-related NMJ transmission failure.

To test whether reduced NaV1.4 function is sufficient to reproduce age-related transmission defects, we acutely inhibited NaV1.4 in adult rats using μ-conotoxin GIIIB, a selective pore blocker of skeletal muscle NaV1.4. Adult rats (3–4 months) received intramuscular injection of μ-conotoxin GIIIB or vehicle in the gastrocnemius. Neuromuscular transmission was assessed using repetitive nerve stimulation (RNS) and stimulated SFEMG.

**Figure 4:**
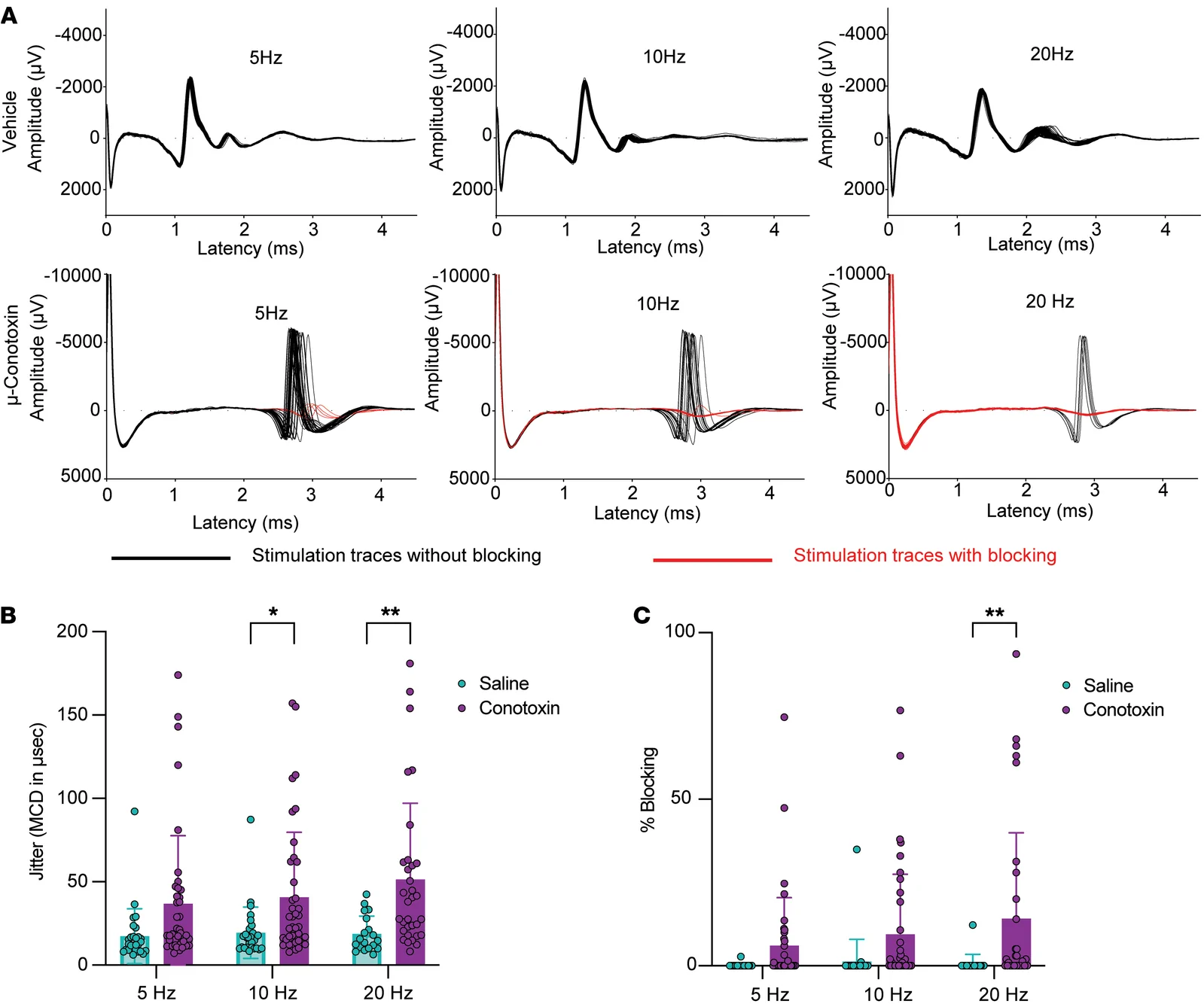
Selective pharmacologic inhibition of NaV1.4 with μ-conotoxin in muscles from adult rats replicates aging-like NMJ transmission defects. (**A**) Representative SFEMG traces recorded at 5 Hz, 10 Hz, and 20 Hz stimulation in saline- (top) and μ-conotoxin–injected (bottom) muscles. μ-Conotoxin markedly increased variability in latency and blocking compared with vehicle. Red traces indicate blocked responses. (**B**) Quantification of jitter demonstrated significant increases in μ-conotoxin–treated rats at 10 and 20 Hz stimulation. (**C**) Percentage blocking was also significantly elevated in the μ-conotoxin group, with effects most pronounced at 20 Hz. Each data point represents an individual fiber measurement. Data are shown as mean ± SEM. Statistical analyses were performed using 2-way ANOVA with Šidák post hoc correction for multiple comparisons. *n* = 3 rats per group (Wistar, 3–4 months old). *P < 0.05; **P < 0.01.

μ-Conotoxin–treated rats demonstrated significantly increased CMAP decrement during RNS, whereas vehicle-treated controls maintained stable responses (Supplemental Figure 5). SFEMG revealed a frequency-dependent increase in jitter and blocking following NaV1.4 inhibition, particularly at 10 and 20 Hz stimulation (*P* < 0.01), recapitulating electrophysiological features observed in aged animals (Figure 4). Thus, acute functional impairment of NaV1.4 is sufficient to induce NMJ transmission failure resembling the aging phenotype, supporting a causal link between reduced junctional NaV1.4 enrichment and age-associated decline in neuromuscular reliability.

### Investigation of ClC-1 inhibition to enhance muscle excitability, NMJ transmission, and muscle function

The findings of NMJ failure in older adults and rodents raised the possibility that treatment to enhance NMJ transmission could offer a useful approach to restore muscle function (e.g., strength) and, in turn, potentially improve mobility and quality of life. Skeletal muscle–specific Cl^–^ (ClC-1) channels are well known to serve as the primary negative regulator of muscle excitability (39), and recent work has reported enhancement of muscle force and NMJ transmission via small-molecule inhibition of ClC-1 channels in primary and secondary NMJ deficits (e.g., myasthenia gravis, pharmacological NMJ disruption, hereditary peripheral nerve disease and denervation [Charcot Marie Tooth Disease]) (40–43). Based on this, we wanted to establish whether small-molecule inhibition of ClC-1 could improve muscle strength in old rats with SFEMG findings of jitter and blocking like those observed in older humans in the clinical study.

#### Characterization of small-molecule inhibitors of ClC-1.

We conducted a series of experiments to characterize 2 small-molecule inhibitors: NMD1226 and NMD653. In this line of experiments we (a) confirmed that ClC-1 function remains intact in old age by showing that resting membrane conductance (G_m_) was the same in muscle fibers from old and adult rats; (b) confirmed that the small-molecule NMD1226 inhibits ClC-1, first, through concentration-dependent reduction in the G_m_ of individual muscle fibers in intact muscles from adult rats and, second, by demonstrating that NMD1226 was able to decrease maximum ClC-1 current in Chinese hamster ovary (CHO) cells expressing ClC-1 channel on an automated patch clamping system; (c) confirmed that the ClC-1 inhibitor NMD1226 increases muscle fiber excitability; and (d) confirmed that NMD1226 restores muscle function in an experimental condition of compromised NMJ transmission (i.e., AChR antagonism via tubocurarine). More details on these results from both NMD1226 and NMD653 are described in the supplemental Methods, Supplemental Figure 6 and Supplemental Table 3.

### ClC-1 chloride inhibition enhances muscle function in aged rodents

#### Old rats exhibit NMJ transmission failure similar to observations from older humans.

**Figure 5:**
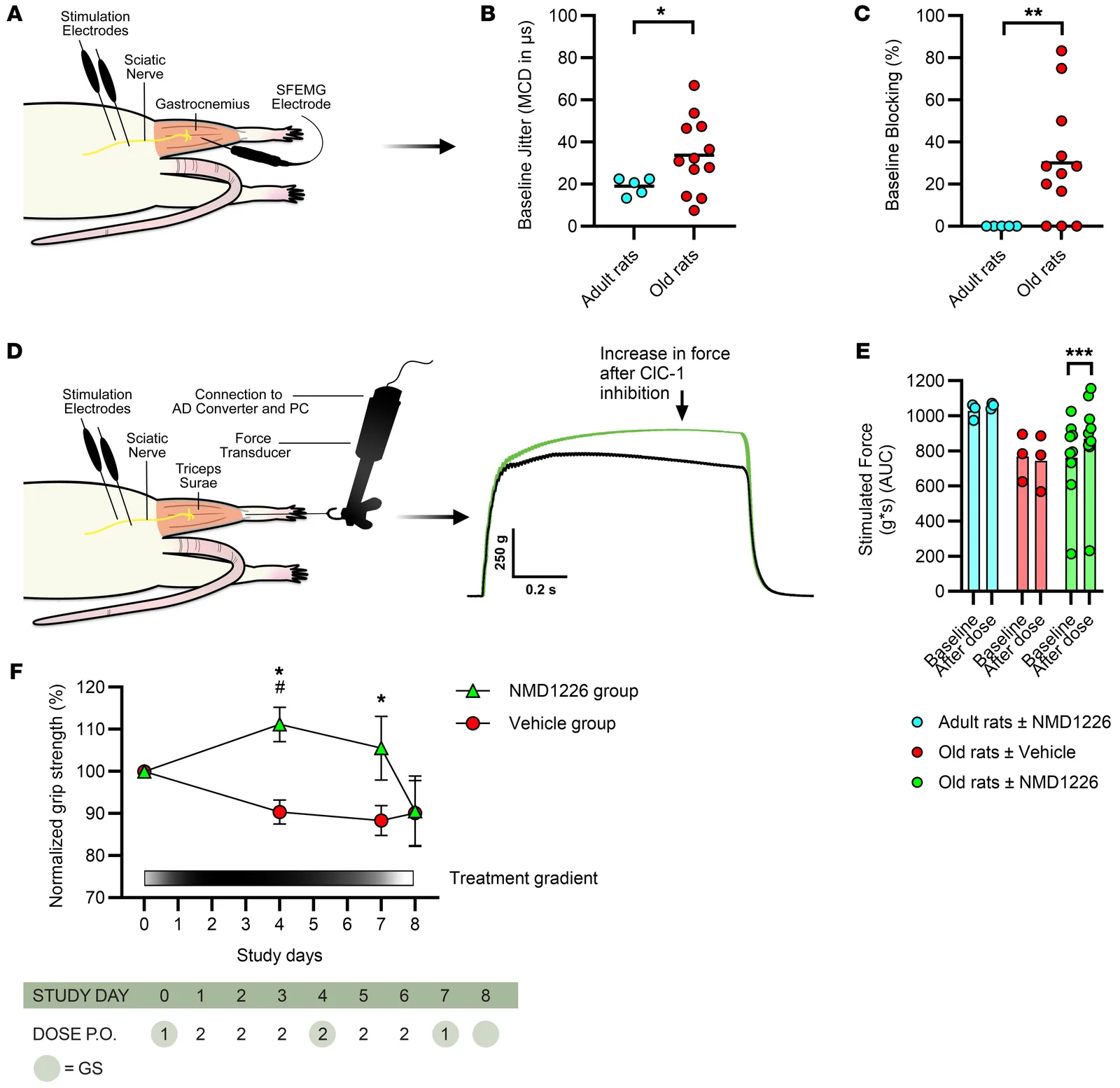
Older rats exhibit impaired NMJ transmission, and ClC-1 inhibition enhances muscle function. (**A**) Schematic of SFEMG measurements in anesthetized rat gastrocnemius muscles. (**B** and **C**) Jitter and NMJ blocking (percentages of synapses with transmission failure) were significantly higher in old rats compared with adult rats (jitter, *P = 0.0176; blocking, **P = 0.003; adult, *n* = 5; old, *n* = 12). Graphs show individual values, mean, and *P* values from *t* tests with Welch’s correction. (**D**) Schematic of nerve-stimulated triceps surae force measurements of anesthetized old rats, with representative 80 Hz force traces before dosing (black) and after 60 mg/kg NMD1226 (green). (**E**) Absolute stimulated muscle force (AUC) at 80 Hz measured before dosing (Baseline) and after NMD1226 or vehicle (After dose). NMD1226 acutely increased force in old rats by 14.4%, whereas force decreased by 3.6% in vehicle-treated old rats and was largely unchanged in NMD1226-treated adult rats (+2.96%), (adult with or without NMD1226, *n* = 3; old with or without vehicle, *n* = 3; old with or without NMD1226, *n* = 9). The graph shows individual values with mean; asterisks show 2-way repeated measures ANOVA (group × treatment interaction *P* = 0.0140) followed by Fisher’s LSD test (***P = 0.0002). (**F**) Grip strength (GS) normalized to body weight was assessed as percentage change from baseline in a blinded 7-day dosing study in 20-month-old rats treated with vehicle (*n* = 4) or NMD1226 (*n* = 5), with day 8 as a predefined washout period of approximately 26 hours. “Dose P.O.” in the timeline indicates the number of oral doses/day. Mean GS decreased by 9.7% in vehicle-treated rats and increased by 11.2% in NMD1226-treated rats from day 0 to 4. Two-way repeated measures analysis of covariance with baseline GS as a covariate showed significant interaction (group × time interaction *P* < 0.01) and effect size (η² = 0.536). LSD post-hoc testing indicated significant increases in GS with NMD1226 on days 4 and 7 (*P = 0.017 and 0.005) and a group difference on day 4 (#*P* = 0.03). Data are shown as mean ± SEM.

Having confirmed the ability of the compounds to inhibit ClC-1, the next series of experiments returned to the core objective of evaluating whether enhancing NMJ transmission can restore muscle function in old animals with confirmed NMJ transmission failure. To this end, we first assessed NMJ function using stimulated SFEMG in old rats (Figure 5A, illustration of SFEMG setup) and confirmed increased jitter and blocking indicative of NMJ transmission failure, similar to prior observations in older adult humans. The old rats demonstrated close to a doubling of jitter (Figure 5B). Most old rats (9 of 12, 75%) showed blocking. In contrast, blocking was not seen in the adult group. On average old rats showed blocking at approximately 30.0% of synapses tested per animal (Figure 5C). These findings closely paralleled the clinical NMJ failure phenotype in older adults and provided a clear rationale to test ClC-1 inhibition in aged rats.

#### ClC-1 inhibition improves NMJ transmission and muscle contractile function in old rodents.

After confirming SFEMG evidence for NMJ transmission failure in old rats, the effect of NMD1226 on muscle force was determined in anesthetized old rats. Rats were anesthetized and placed in an experimental setup that enabled recordings of contractile force from hind leg muscles in response to stimulation of the sciatic nerve (Figure 5D). At baseline, compared with adult rats (adult rats, 7.1 ± 0.9 months, 618 ± 98 grams, *n* = 3; vs. old rats, 22 ± 2 months, 668 ± 86 grams, *n* = 12), the force production during sustained contraction (1 s, 80 Hz stimulation) was 26% lower in the old rats (1,028 g × s vs. 764 g × s) (Figure 5E). As exemplified by the black trace in Figure 5D, it was furthermore observed that force production tended to decline during the 1-second train of 80 Hz stimuli in old rats. However, oral administration of NMD1226 increased force production and reduced the decline during the contraction (Figure 5D, green trace). Force enhancement was observed in all old rats, and the average force increase was 14% (average baseline force of 762 g × s increased to 870 × s after dosing, Figure 5E). Accordingly, ClC-1 inhibition resulted in rescue of >50% of the force deficit seen in old versus adult rats. In contrast, old rats treated with vehicle showed a slight reduction of force production (Figure 5E). Furthermore, ClC-1 inhibition showed no change in force production in the adult rats that did not present with NMJ transmission deficits on SFEMG (Figure 5E). Collectively, these observations suggested that ClC-1 inhibition can improve muscle function in old rat model with detectable NMJ transmission deficit.

#### ClC-1 inhibition improves grip strength in old rats.

After observing restored force production in old rats in response to nerve stimulation, we were interested in understanding the effect that ClC-1 inhibition could have on voluntary motor function. To explore this, we conducted a blinded study in which old rats were treated with either NMD1226 or vehicle twice daily over 7 days. On day 0, rats underwent grip strength testing to evaluate voluntary muscle strength prior to treatment, and then grip strength was retested while on treatment (or vehicle) on days 4 and 7. At study end on day 8, grip strength was reevaluated when animals were taken off treatment (see insert in Figure 5F). At baseline (prior to treatment), the NMD1226 group exhibited numerically lower grip values, but this was not statistically significant (NMD1226 group, 1.84 ± 0.11 g/g BW; vehicle group: 2.13 ± 0.06 g/g BW, *P* = 0.1833). During the treatment period, the aged rats treated with the NMD1226 showed an increased grip strength (day 4 and 7) compared with a gradual decline in grip strength in the vehicle treated rats (Figure 5F). The positive effect of NMD1226 on grip strength was completely abolished following removal of treatment as grip strength reverted and became almost identical to that of the control group 1 day after terminating administration of NMD1226 (day 8). In a complementary experiment, aged C57BL/6 mice underwent baseline RNS assessment and were then randomized to receive acute dosing of a ClC-1 inhibitor (NMD653) (*n* = 11 [6 females, 5 males], mean age, 27.9 months; range, 27–29 months) or vehicle (*n* = 11 [4 females, 7 males], mean age, 28.3 months; range age, 27–29 months). Following treatment, mice underwent RNS for CMAP decrement as well as terminal SFEMG. Treatment with ClC-1 inhibition resulted in significantly improved function compared with vehicle treatment on RNS and mean SFEMG jitter (NMD653, 13.66 ± 5.26 μs vs. vehicle, 20.42 ± 8.93 μs), indicating improved NMJ transmission and aligning with findings in aged rats (Supplemental Figure 7).

## Discussion

Here, through integrated human and animal investigations, we demonstrated that age-related NMJ transmission deficits coincide with a loss of NaV1.4 channels at the postsynaptic membrane and likely play a meaningful role in age-related neuromuscular dysfunction. Together, these data provide a biologically grounded rationale for evaluating ClC-1 inhibition as a potential approach to improve NMJ transmission and muscle performance in older adults. We first demonstrated that NMJ transmission is impaired in weak older adults and that NMJ transmission deficits are correlated with muscle strength (normalized to muscle volume). Next, we identified a localized reduction of excitability associated with a reduced density of NaV1.4 channels at the NMJ postsynaptic membrane in aged rodents, suggesting loss of postsynaptic muscle membrane excitability as a key mechanism of NMJ deterioration in aging. To link this finding to humans, we demonstrated that older adults exhibit similar losses of NaV1.4 channel density at the NMJ postsynaptic membrane, indicating a potential mechanism behind NMJ transmission failure in aging. We then showed that inhibiting NaV1.4 channels with μ-conotoxin GIIIB in adult rats reproduced the SFEMG evidence of NMJ transmission failure seen in aged rodents and humans. Finally, in aged rodent models with SFEMG-defined NMJ dysfunction similar to that observed in weak older adults, small-molecule ClC-1 inhibition restored muscle contractile function during both stimulated and voluntary contractions and improved electrophysiological measures of NMJ transmission. These results position NMJ transmission as a key and potentially reversible contributor to sarcopenia within a multifactorial framework of age-related muscle dysfunction, supporting it as a promising therapeutic target.

Current sarcopenia treatments mainly include exercise interventions and optimizing nutrition (44). However, the responses to these interventions vary widely (45–47), and adherence can be challenging (48, 49), leaving a gap for therapeutic approaches. In the past two decades, several function-promoting therapies have been investigated, mostly centered on anabolic strategies to increase muscle mass (e.g., myostatin inhibitors) (50). Unfortunately, these trials have shown only modest improvements in muscle function (44). The potential of targeting NMJ transmission for sarcopenia has remained largely unexplored. Our identification of NMJ transmission defects in weak older adults highlights the NMJ as a therapeutic focus area.

### Age-related loss of NMJ form and function.

There has been enduring interest in understanding age-related changes at the NMJ (17, 18, 51). Previous studies have primarily focused on assessing NMJ morphology, revealing that aged preclinical rodent models often exhibit NMJ fragmentation and denervation (17, 18, 32, 51–53). As morphological studies of human NMJs present more varied results due to challenges in obtaining muscle samples, it remains uncertain whether aging affects NMJ morphology similarly in rodents and humans (38, 54, 55). Data obtained from dorsal interosseous muscles in the current study, however, strongly support a model whereby NMJs remain structurally intact over the human lifespan, with denervation being a feature of aging in rodents but not in humans. The knowledge gap on how aging affects NMJ morphology is, however, surpassed by the lack of understanding of how aging affects NMJ function. For instance, no studies have directly assessed NMJ transmission across the lifespan in either humans or rodents. Notably, in rodent models, isolated EP have frequently shown an increase in EP responses (e.g., potentials or currents) in aged animals, suggesting a lack of overt NMJ failure in old age (32, 56, 57). While this appears to be at odds with any age-related failure of NMJ transmission, it is important to consider that these synaptic potentials are not complete evaluations of NMJ transmission. To accurately assess NMJ transmission, it is crucial to determine whether these synaptic potentials trigger muscle fiber APs.

Therefore, we utilized the stimulated SFEMG technique to directly evaluate NMJ transmission efficacy, providing electrophysiological evidence (increased jitter and blocking) indicating NMJ dysfunction and failure in clinically meaningful, age-related weakness. Within our clinical cohort, we identified failure in up to 35% of synapses per older adult (range, 0%–35%). Similarly, in the preclinical aspect of the study, we observed increased jitter and blocking in old rats (with an average increase in blocking observed in 30% of synapses), closely resembling our clinical findings. This collective observation reveals consistent NMJ transmission failure in aging across species, demonstrating a relatively similar magnitude and variability of synaptic failure.

SFEMG findings of blocking indicate a failure of an endplate response to trigger an AP, resulting in reduced muscle activation and force production. Accordingly, our preclinical and clinical findings suggest that NMJ dysfunction could have major negative consequences on muscle function. In contrast, jitter does not indicate failure of NMJ transmission but increased variation of AP timing (latency). While some jitter is normal, heightened jitter suggests synaptic abnormality that precipitates blocking. It is important to note that the parameter of near-fiber “jiggle,” obtained through computer-automated decomposition-based quantitative EMG approaches, shares some similarities with SFEMG jitter (58–60). However, jiggle measures motor unit variability or stability between discharges, while SFEMG assesses the timing of individual muscle fiber potentials (58). This difference could explain the discrepancies in prior studies using decomposition-based quantitative EMG approaches to evaluate NMJ transmission in older adults (23–26).

After confirmation of NMJ transmission failure in older adults, we investigated the mechanism of this failure. Our findings suggest a potential mechanism for age-related muscle weakness by focusing on the localized alterations of excitability at the NMJ, occurring in the absence of any overt structural changes or denervation in human NMJs. Given previous observations of maintained or even increased EP size in aged rodents, we hypothesized a postsynaptic mechanism contributing to age-related NMJ transmission failure. While prior reports in aged rodent muscle have shown altered excitability in aged rodents (61–63), we found no evidence of such global deficits in isolated EDL fibers. Across multiple electrophysiologic measures, including resting membrane potential, input resistance, time constant, and sustained AP firing, fibers performed similarly in adult and old mice, indicating preserved whole-fiber sarcolemmal excitability. After confirming that there was no loss of excitability at the whole muscle fiber level, we directed our investigation toward changes localized to the NMJ, revealing that a higher proportion of muscle fibers in older mice and rats exhibited a lack of larger junctional excitability than extrajunctional excitability compared with their younger counterparts. Notably, we identified a parallel, significant age-related reduction in NaV1.4 channel expression level at the NMJ in both aged rodents and humans.

The decline in NaV1.4 expression was muscle dependent, evident in fast-twitch muscles such as the EDL and sternomastoid but not in the slow-twitch soleus. This suggests that NMJs in fast glycolytic muscles are particularly vulnerable to aging, aligning with the preferential atrophy and early loss of type II fibers reported in sarcopenia (64, 65). Importantly, NaV1.4 transcript levels were largely preserved in the EDL, and we found no evidence of redistribution of NaV1.4 along the sarcolemma, indicating that the age-related reduction primarily reflects localized altered protein maintenance or localization rather than decreased gene expression at the whole muscle level. Together, these findings suggest a muscle- and fiber-type–specific postsynaptic mechanism in which reduced NaV1.4 channel availability at the NMJ diminishes the safety factor for transmission and contributes to age-related NMJ failure. The upstream mechanisms responsible for age-related reductions in NaV1.4 and postsynaptic excitability are not yet clear, but the MuSK/BMP signaling pathway represents a plausible contributor (66). Prior studies show that perturbing this pathway in adult mice recapitulates the key features we identified, including diminished NaV1.4 at the NMJ and impaired transmission, which raise the possibility that age-related shifts in MuSK/BMP signaling may underlie the phenotype observed in our human and animal data (66).

To establish a direct functional link between NaV1.4 activity and NMJ transmission fidelity, we acutely inhibited NaV1.4 channels in adult rats using the selective blocker μ-conotoxin GIIIB. This transient reduction in NaV1.4 function reproduced the defining electrophysiological signature of aging-related NMJ failure, producing increased jitter and impulse blocking during SFEMG recordings. These findings provide direct evidence that loss of NaV1.4 activity is sufficient to destabilize NMJ transmission. Together with prior genetic mouse studies showing that partial NaV1.4 deficiency produces fatigable weakness and that selective loss of NMJ-localized NaV1.4 in ankyrin-deficient mice causes NMJ transmission failure (67, 68), our findings further support NaV1.4 as a critical determinant of postsynaptic excitability and transmission reliability. Moreover, a congenital myasthenic syndrome caused by NaV1.4 dysfunction has been reported, providing additional human evidence for its essential role at the NMJ (69). Taken together, these converging lines of evidence suggest that age-related NaV1.4 decline is not merely correlative but may represent a mechanistic driver of NMJ transmission failure and, by extension, a viable therapeutic target for mitigating sarcopenic weakness. Importantly, our findings reveal two unexpected insights: first, that NMJ failure may play a crucial role in sarcopenia, a factor not commonly recognized in clinical settings. Second, the underlying mechanism stems from a loss of gain of the synaptic signal by the muscle fiber, highlighting a postsynaptic dysfunction that has previously been overlooked.

### ClC-1 channel inhibition to promote function in sarcopenia.

In a recent study on myasthenia gravis, inhibition of the ClC-1 channel was found to enhance NMJ transmission and improve muscle function in both animal models and patients with myasthenia gravis (41). Our current findings demonstrate that small-molecule inhibition of ClC-1 can similarly enhance muscle contractile function and improve motor function assessments in aged, weak rats with confirmed NMJ dysfunction. The ClC-1 Cl^–^ ion channel is specific to skeletal muscle, expressed along the entire muscle fiber membrane, including at NMJ, sarcolemma, and t-tubular system. This channel plays a critical role in modulating skeletal muscle excitability, especially during intense muscle activity, through cellular signaling systems activated during muscle activity (70). While not yet directly measured at the NMJ, this ClC-1 regulation could also be occurring there, potentially contributing to NMJ transmission function during repeated AP firing. Age-related alteration of coupling between Cav1.1 calcium channels and ryanodine receptors has been proposed as a contributor to reduced muscle fiber–specific force (71). By heightening muscle fiber excitability, ClC-1 inhibition may also strengthen t-tubular depolarization and partially compensate for age-related EC-coupling deficits, supporting more reliable Cav1.1-RyR1 activation during repeated stimulation and contributing to the improved contractile output we observed. However, this remains inferential, as EC-coupling dynamics and Ca²^+^ transients were not directly assessed in aged muscle, and future studies will be needed to confirm this mechanism.

The development of new therapies for sarcopenia faces challenges due to the diverse and variable nature of contributing mechanisms underlying weakness. Some theories suggest that distinct phenotypes of sarcopenia exist, possibly requiring different treatment approaches (72–74). NMJ transmission defects offer a well-defined biological pathway with clear clinical implementation potential. Using NMJ transmission measures as physiological biomarkers could aid in diagnosing sarcopenia, classifying its subtypes, stratifying between treatment regimens (e.g., identifying individuals likely to respond to an NMJ-targeted therapeutic), and assessing target effectiveness.

Exploring NMJ transmission phenotypes in sarcopenia has the potential to expedite treatment development and adoption. The potential impact of a muscle function promoting therapy is substantial considering the costly burden of sarcopenia on healthcare systems. Muscle dysfunction increases risk of falls and fractures, impedes daily activities, causes mobility issues, and leads to diminished quality of life, loss of independence, or the need for long-term care, and even mortality (7). The relevance of treatments to improve muscle function in sarcopenia is thus very clear. Future studies comparing sarcopenic and nonsarcopenic older adults will be important to define how NMJ transmission abnormalities relate to clinical weakness and functional decline.

### Conclusion.

Sarcopenia is a complex disorder associated with multiple pathophysiological factors that contribute to onset, progression, and severity of muscle function decline. This study offers direct clinical evidence for NMJ transmission deficits contributing to weakness in older adults. Additionally, we identified a localized loss of excitability at the postsynaptic NMJ membrane in aged individuals, associated with a reduced density of NaV1.4 channels. This finding highlights a central mechanism underlying NMJ transmission failure in both aged rodents and humans. Importantly, this excitability defect is distinct from synaptic cholinergic transmission abnormalities and occurs independently of denervation, underscoring a mechanism contributing to sarcopenia. Our preclinical studies highlight the potential of small-molecule inhibition of the ClC-1 channel as a promising avenue for enhancing NMJ transmission in clinical trials among older adults with weakness. These collective findings strongly support the future development and application of therapies targeting NMJ transmission to improve muscle function in older adults.

## Methods

A detailed description of the experimental procedures and reagents and material sources, instrument details, and technical specifications is provided in the Supplemental Methods.

### Sex as a biological variable

In the clinical dataset, both male and female participants were included, with balanced representation of the two groups. For most of the preclinical experiments, except where indicated, only male animals were used. This was done to ensure consistency across all assessments and study sites, as aged male animals were more readily available across the collaborating sites.

### Clinical assessment of NMJ transmission

#### Study design.

The STAMINA study (NCT04904926) compared healthy young and middle-aged adults (18–50 years) to older adults (70+ years) with age-related weakness. Assessments included leg extension isokinetic strength at 60°/s, physical function, quadriceps MRI, and NMJ transmission by SFEMG. Physical function/mobility was assessed using the composite short physical performance battery score, stair climb power, and four-square step test time, as previously described (75, 76).

Quadriceps femoris volume was assessed using magnetic resonance imaging (MRI) as described previously (77). Percentage body fat and appendicular lean mass relative to height^2^ were quantified using dual-energy X-ray absorptiometry.

We assessed NMJ transmission in the left vastus lateralis with stimulated SFEMG using a Sierra Summit system (Cadwell) according to the published guidelines (59). Suitable single-fiber APs were identified, and stimulation intensity was adjusted to ensure supramaximal axonal stimulation. A total of 50–100 consecutive stimulations at 10 Hz were analyzed at each synapse for jitter and blocking. Responses with jitter <5 μs were excluded to avoid possible split muscle fibers or direct muscle stimulation.

### Muscle fiber and NMJ excitability in rodents

#### Isolated single-muscle fiber excitability in aged mice.

EDL muscles from aged mice were dissected, maintained in Ringer solution, stained with 4-Di-2ASP, and recorded using 2 electrodes within 6 hours after dissection. APs were evoked by current injection, and membrane properties were measured using hyperpolarizing current steps.

#### Junctional and extrajunctional excitability in aged mice and rats.

EDL muscle fibers were imaged and impaled near (junctional) and far from (extrajunctional) NMJs using 2 (mouse) or 3 (rats) sharp electrodes. The current injection duration varied between 0.2 and 1 ms, and the AP triggering charge amount was compared at junctional versus extrajunctional sites. The excitation current threshold was determined by progressively increasing the stimulation current until eliciting an AP.

Localized postsynaptic excitability “gain” was defined as requiring less stimulation to trigger an AP at the NMJ than in extrajunctional regions. Each fiber was classified dichotomously as exhibiting gain, junctional-to-extrajunctional rheobase ratio <1.0, or not. Using a binary measure aligns with the all-or-none nature of NMJ transmission and provides a statistically robust metric at the single NMJ level for comparing fibers that retain the gain for reliable NMJ activation.

### Preclinical and clinical assessment of NaV1.4 channel density at the NMJ

#### Rodent NMJ immunofluorescence staining.

Rodents were euthanized, and sternomastoid and EDL muscles (mice) or lumbrical muscles (rats) were dissected, fixed, and immunolabeled for NaV1.4 and AChRs. Additionally, mouse tissue was stained for muscle fiber–type myosin heavy chains. Stained NMJs were imaged by confocal microscopy, with 23–30 NMJs analyzed per animal. NaV1.4 and AChR signals were quantified using Fiji/ImageJ, and NaV1.4 area was normalized to NMJ size. Antibody sources, catalog/clone numbers, and reagent details are provided in the Supplementary Methods.

#### Human NMJ sample preparation, staining and imaging.

Human muscle biopsy samples were fixed and processed for teased fiber immunohistochemistry and immunolabeling for presynaptic markers (3A10/SV2), Nav1.4, and AChRs. NMJ imaging and gross morphological analyses were performed using the “NMJ-morph” workflow on a Zeiss LSM 710 confocal microscope to assess 21 morphological variables, with 40 NMJs analyzed per muscle. Muscle fiber diameter was measured using ImageJ (NIH), with 40 fibers analyzed per muscle. Antibody/reagent sources, catalog/clone numbers, and staining details are provided in Supplemental Methods.

To measure parajunctional NaV1.4 rim width, confocal micrographs (*z* stack maximum intensity projection of both NaV1.4 and BTX signal) of individual, en face NMJs were analyzed using ImageJ (NIH). For comparative analysis of NaV1.4 area between adult and old human NMJs, a nearest-neighbor matching algorithm was applied to pair NMJs based on similarity in AChR area. Pairs differing by more than 10% in AChR area were excluded, resulting in 34 matched NMJ pairs included in the final quantitative analysis. Because each older NMJ was explicitly matched 1:1 to an adult NMJ based on AChR area (≤10% difference) and the resulting dataset consisted of dependent paired observations rather than independent samples, within-pair differences were therefore analyzed using a paired 2-tailed *t* test (78, 79).

#### SFEMG recordings following μ-conotoxin administration.

Adult male Wistar rats (3–4 months old, *n* = 3 per group) received intramuscular injections of μ-conotoxin GIIIB (166 ng/g; Alomone Labs) or saline into the gastrocnemius muscle. RNS was used to assess CMAP decrement, calculated as the percentage decrease between the first and tenth CMAP during 50 Hz stimulation. Forty minutes after injection, stimulated SFEMG was performed by an investigator blinded to the treatment groups to quantify jitter and blocking in the gastrocnemius muscle at 5, 10, and 20 Hz. Jitter and blocking were evaluated from 50–100 stimulations per synapse and compared between vehicle- and μ-conotoxin–treated rats. Detailed procedures are provided in the Supplemental Methods.

### Investigation of ClC-1 inhibition to enhance NMJ transmission and muscle function

G_m_ and rheobase were measured in isolated soleus fibers from 3-month-old female Wistar rats (RjHan:WI, Janvier Labs) to evaluate the ClC-1 inhibitory effects of NMD1226 and NMD653 using methodology described in detail elsewhere (80). Detailed procedures for the following ClC-1 inhibition procedures are provided in the Supplemental Methods.

#### ClC-1 currents measured in CHO cells.

An automated patch clamp system, Qpatch I system (Sophion Bioscience A/S), was used to estimate the effect of the small molecules NMD1226 and NMD653 on ClC-1 currents in CHO cells stably expressing the human isoform of ClC-1.

#### Force measurement in isolated intact nerve-muscle preparations.

Ex vivo whole muscle force was measured in soleus muscles from 1-month-old Wistar rats of mixed sex (50:50 male/female) that were mounted on force transducers and stimulation via nerve and direct muscle fiber stimulation. Force was depressed with 0.115 μM tubocurarine, and after 90 minutes, 50 μM of either NMD1226 or NMD653 was added, leading to a steady recovery of force. This setup demonstrated the potential of ClC-1 inhibition to enhance muscle force generation under neuromuscular blockade (40).

#### Preclinical stimulated single fiber EMG.

Adult and aged rodents underwent stimulated SFEMG assessment of jitter and blocking of the gastrocnemius using a Sierra Summit system (Cadwell) (59). Rodents were anesthetized with isoflurane and maintained at 37°C, and single-fiber APs were elicited by 10 Hz sciatic nerve stimulation. Jitter was averaged per animal, and blocking was reported as the percentage of synapses exhibiting transmission failure.

#### Stimulated muscle force in anesthetized rats.

Stimulated triceps surae force was measured in anesthetized, mechanically ventilated rats as previously described (81). The Achilles tendon was attached to a force transducer, and sciatic nerve stimulation was used to elicit contractions. After force stabilization, 60 mg/kg NMD1226 was administered by oral gavage, and changes in force responses were monitored. Muscle force from 80 Hz stimulation was quantified as AUC.

#### 7-day blinded preclinical interventional study.

A 7-day blinded study evaluated the effects of 60 mg/kg NMD1226 (5 rats) and vehicle (sterile water, 4 rats) via oral gavage twice daily on 20-month-old male Wistar rats. Grip strength tests were performed at baseline (prior to treatment), day 4 (on drug or vehicle), day 7 (on drug or vehicle), and day 8 (after washout) to assess muscle strength. Valid attempts were averaged for analysis, and data analysis was completed before unblinding.

#### Acute ClC-1 inhibition in aged mice.

Aged C57BL/6 mice underwent RNS and were randomized to receive a single intraperitoneal dose of NMD653 (20 mg/kg) or vehicle, followed by RNS and terminal stimulated SFEMG. Sciatic nerves were stimulated supramaximally and recorded from the gastrocnemius to assess CMAP amplitude and percentage decrement during a train of 10 stimulations delivered at 10–50 Hz before and after intraperitoneal dosing. SFEMG was performed following dosing to quantify jitter and blocking during 10 Hz stimulation (20, 82). Electrophysiologic analyses were conducted by an investigator blinded to treatment groups.

### Statistics

Statistical analyses were prespecified and performed using SPSS Statistics version 27 (IBM) and GraphPad Prism (GraphPad Software). Data distributions were assessed and parametric or nonparametric tests were selected accordingly. All statistical tests were 2-tailed, and a preset α level of 0.05 was required for statistical significance. Data are presented as individual values or as mean ± SEM or SD, as indicated, and *n* values represent the number of animals, muscles, experiments, or human participants analyzed. The details for statistical analyses are provided in the Supplemental Methods.

### Study approval

#### Clinical.

The clinical cross-sectional SFEMG study (STAMINA; NCT04904926) was conducted with approval from The Ohio University Institutional Review Board (IRB 20-F-17). The clinical study investigating NMJ morphology in human samples was conducted with approval from the NHS Grampian Ethics Committee (REC reference: 20/NS/0008; protocol: AC18077; IRAS project ID: 244717). Written informed consent was obtained from all participants prior to their involvement.

#### Mice.

Terminal NMJ imaging analyses were conducted in mice at the University of Missouri with approval from the Animal Care Quality Assurance Office of the University of Missouri (protocol 39905). Terminal electrophysiological assessment of junctional and extrajunctional excitability was conducted in mice at Wright State University with approval from Wright State University Institutional Animal Care and Use Committee (protocol 2023-0139). Acute ClC-1 inhibition in aged mice to assess CMAP, RNS, and SFEMG was conducted in mice at The Ohio State University in accordance with the approval of Institutional Animal Care and Use Committee of The Ohio State University (protocol 2018A00000005).

#### Rats.

For preclinical studies in rats, all animal experiments were performed in compliance with Danish Animal Welfare regulations and approved under license 2020-15-0201-00402, or performed in accordance with ethical protocols approved by the Institutional Animal Care and Use Committee at the University of Missouri (protocol 39905) and were performed in accordance with the NIH *Guide for the Care and Use of Laboratory Animals* (National Academies Press, 2011). Animals were housed in ventilated racks with controlled temperature (20°C–22°C) and humidity (45%–65%), maintained on a 12-hour-light/dark cycle, and provided ad libitum access to food and water. Male and female animals were housed separately, and all procedures, including euthanasia, adhered to approved ethical standards. These measures ensured adherence to ethical standards for human and animal research.

### Data availability

All data and materials are available upon reasonable request of the authors. Supporting data values underlying the data presented in the figures are provided in the Supporting Data Values file.

## Author contributions

Conceptualization: WDA, JJM, MBL, TG, MS, LAF, DC, JB, JQ, JH, JRF, THG, MMR, THP, and BCC. Methodology: WDA, JJM, PBT, MBL, LAC, TG, MS, JBW, LAF, DC, HN, RAJ, XW, JRF, THG, MMR, THP, and BCC. Investigation: WDA, JJM, PBT, LAC, JBW, AR, PAR, JHM, FBD, ARD, HN, RAJ, XW, THG, MMR, and BCC. Visualization: WDA, JJM, PBT, MBL, LAC, TG, MS, AR, PAR, JHM, FBD, ARD, JB, JQ, JH, HN, RAJ, XW, THG, MMR, THP, and BCC. Funding acquisition: WDA, AR, RAJ, THG, MMR, and BCC. Writing of the original draft: WDA, JJM, TG, THP, and BCC. Review and editing of the manuscript: All authors.

## Conflict of interest

The studies were sponsored in part by NMD Pharma. JJM, PBT, TG, MS, MBL, JB, JH, JQ, JBW, and THP were or are employed by NMD Pharma and may own and/or hold options or restricted stock units for the company and/or patents on the designed compounds. WDA has received grant funding, consulting fees, and travel support from NMD Pharma. LAF and JRF are coinventors on patents (63/162,796; 63/197,842; 64/079,979) held by Brown University covering manipulation of the MuSK/BMP pathway, and JRF is a cofounder of Bolden Therapeutics, which has licensed these patents. THG has provided advisory services for Roche, Novartis, and LifeArc.

## Funding support

This work is the result of NIH funding, in part, and is subject to the NIH Public Access Policy. Through acceptance of this federal funding, the NIH has been given a right to make the work publicly available in PubMed Central.

- NIH grants R01AG078129 and R01AG067758 (to WDA and BCC).
- NIH grant AR074985 (to MMR).
- Funding from Anatomy@Edinburgh, University of Edinburgh (to THG and RAJ).
- PhD studentship from King Saud Abdulaziz University for Health Sciences, through the Saudi Cultural Bureau, London (to AR and THG).
- NMD Pharma funded the clinical cross-sectional SFEMG study (STAMINA; NCT04904926) (to WDA and BCC).

## Supplementary Material

Supplemental data

Supporting data values

## Figures and Tables

**Table 2:**
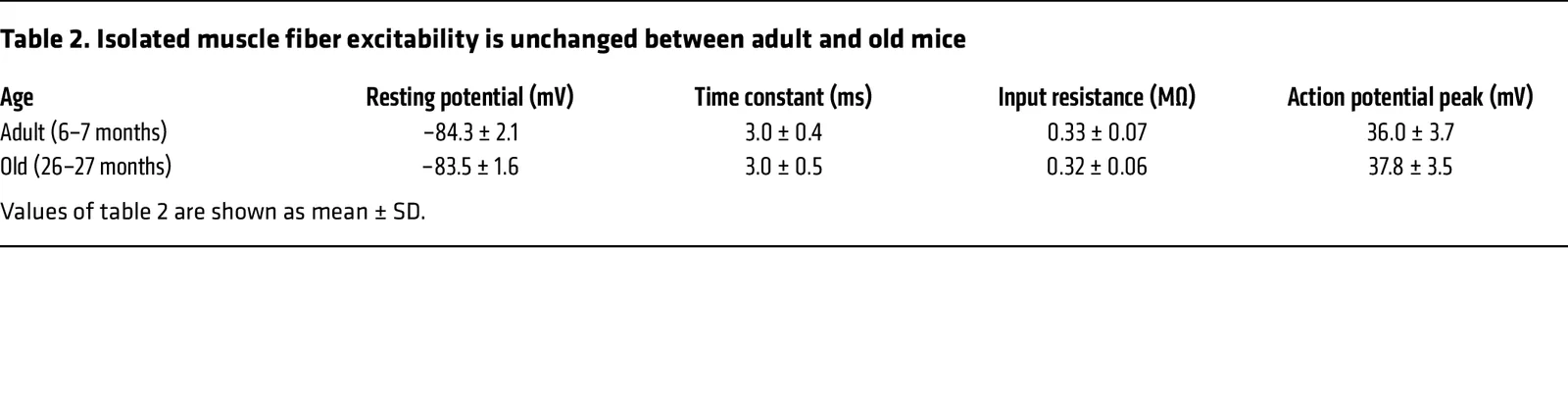
Isolated muscle fiber excitability is unchanged between adult and old mice

**Table 1:**
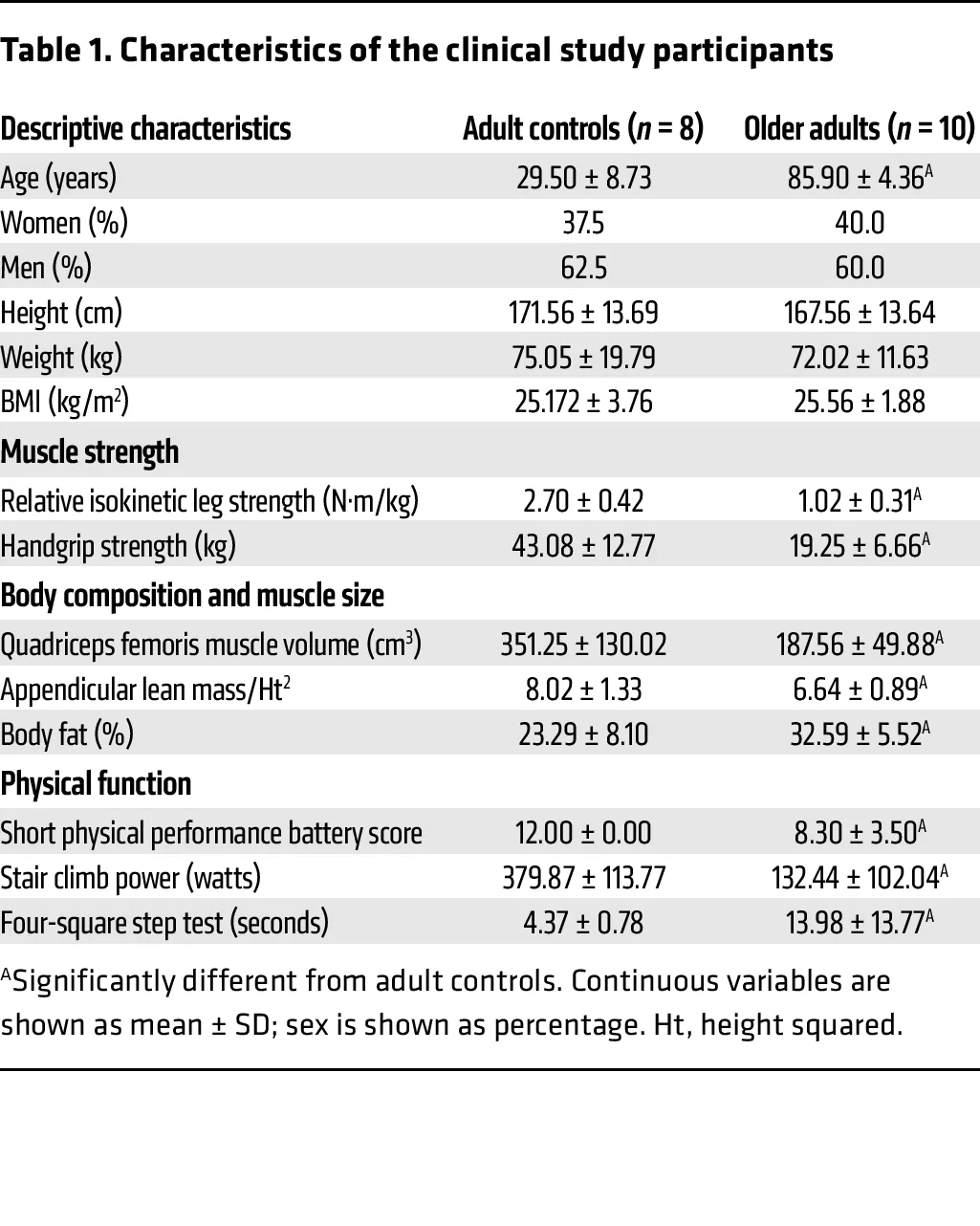
Characteristics of the clinical study participants

## Version 1 07/09/2026

In-Press Preview

## Version 2 09/01/2026

Electronic publication
