# Supplementary material for: Neuromuscular junction failure in sarcopenia is linked to NaV1.4 loss and reversed by ClC-1 inhibition
Source: J Clin Invest. 2026 Jul 9;136(17):e190646. doi: 10.1172/JCI190646 (PMC13528926; doi:10.1172/JCI190646)
Supplement: Supplementary file 1 [file jci-136-190646-s258.pdf]

SUPPLEMENTARY TABLES AND FIGURES AND METHODS

Supplementary Table 1. Inclusion and exclusion criteria for the clinical study on NMJ transmission.

Inclusion Criteria

- Men and women 70+ years of age (Older Adults) OR men and women 18-50 years old (Adults).
- Body mass index between 19 and 40 kg/m^2.
- Self-report physical function limitations.
- Self-report being fully vaccinated for COVID-19 as per CDC definition.
- Willingness to undergo all testing procedures, maintain current diet during the study period, and adhere to the study protocol.

Exclusion Criteria

- Neuromuscular Disease (e.g., movement disorder, or overt neurological disease, such as Sensory Neuropathy with Sensory Ataxia, Apraxia, Post-Polio Syndrome, Mitochondrial Myopathy, Myelopathy, myasthenia gravis)
- Neurological Disease (e.g., Dementia (Alzheimer's, multi-infarct, fronto-temporal); multiple sclerosis, amyotrophic lateral sclerosis, Parkinson's Disease, cerebellar ataxia, or significant cognitive impairment (a score of 22 or less on the Montreal Cognitive Assessment (MOCA).
- Musculoskeletal disorders (e.g., rheumatoid arthritis, ankylosing spondylitis, psoriatic arthritis, acute gout or osteoarthritis limiting mobility).
- Terminal illness (e.g., Cancer, myeloma, acute leukemia).
- Uncontrolled Psychiatric disorder (e.g., bipolar, schizophrenia, major depression)
- Severe Cardiovascular Diseases (e.g., NYHA Class III or IV congestive heart failure, clinically significant aortic stenosis, recent history of cardiac arrest, uncontrolled atrial fibrillation, use of a cardiac defibrillator, or uncontrolled angina or hypertension).
- Other significant conditions that in the investigator's opinion would impact safety and/or compliance to the protocol (e.g., chronic renal failure requiring peritoneal or hemodialysis, chronic liver disease, blood dyscrasia, carcinoma within last 3 years, severe inflammatory bowel disease, severe respiratory disease (uncontrolled asthma, COPD), etc.).
- History of drug or alcohol abuse.
- Not meeting MRI eligibility (e.g., metal implants, reported pregnancy or positive pregnancy test at the time of imaging for women in the control group).
- Not meeting the DEXA eligibility (e.g., reported pregnancy or positive pregnancy test at the time of imaging for women in the control group.
- Failure to provide informed consent.
- Subjects who do not answer "male" or female" to the question of their biological sex assignment.
- Currently or recently (within the last 1 year) taking gender affirming hormones.

Supplementary Table 2. Demographic details of the human case series obtained for NMJ morphology experiments.

Case Number	Age (Years)	Sex	Muscle	# of Assembled Series NMJ	# of Assembled Series Nav 1.4
**14**	19	F	Dorsal interosseous	40 per biopsy	20 per biopsy
**15**	82	F	Dorsal interosseous	40 per biopsy	20 per biopsy
**23**	18	M	Dorsal interosseous	40 per biopsy	21 per biopsy
**27**	23	M	Dorsal interosseous	40 per biopsy	19 per biopsy
**38**	84	F	Dorsal interosseous	40 per biopsy	14 per biopsy
**50**	74	M	Dorsal interosseous	40 per biopsy	13 per biopsy

Biopsies were collected from otherwise healthy muscle from both male and female patients undergoing upper limb reconstructive surgery. The total number of patients was six, and they were divided into two distinct age groups: a Adult Group (18, 19, and 23 years old), and an Old Group (74, 82, and 84 years old).

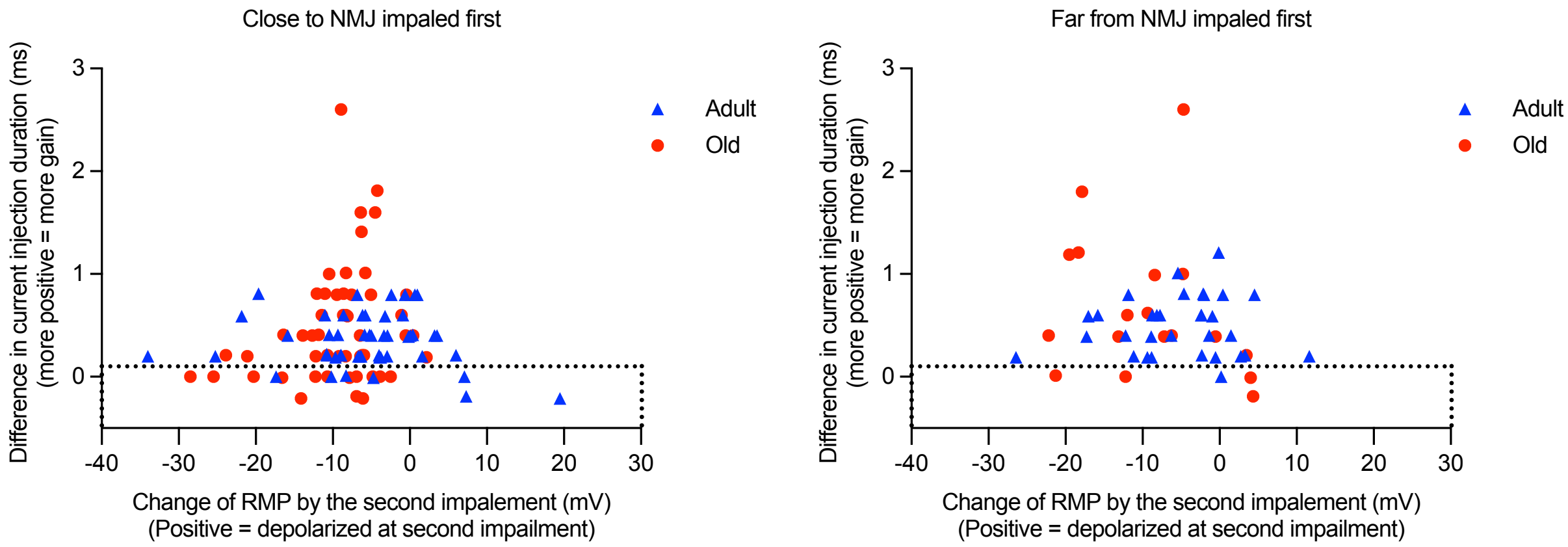

Supplementary Figure 1: The box at the bottom of the plots identifies the subset of fibers that demonstrated lack of synaptic gain. Regardless of the order of impalement and the loss of resting potential with repeated impalement, the majority of fibers with low synaptic gain are from old mice. Note that there is no clear correlation between synaptic gain and loss of resting membrane potential with repeated impalement. NMJ, neuromuscular junction; RMP, resting membrane potential.

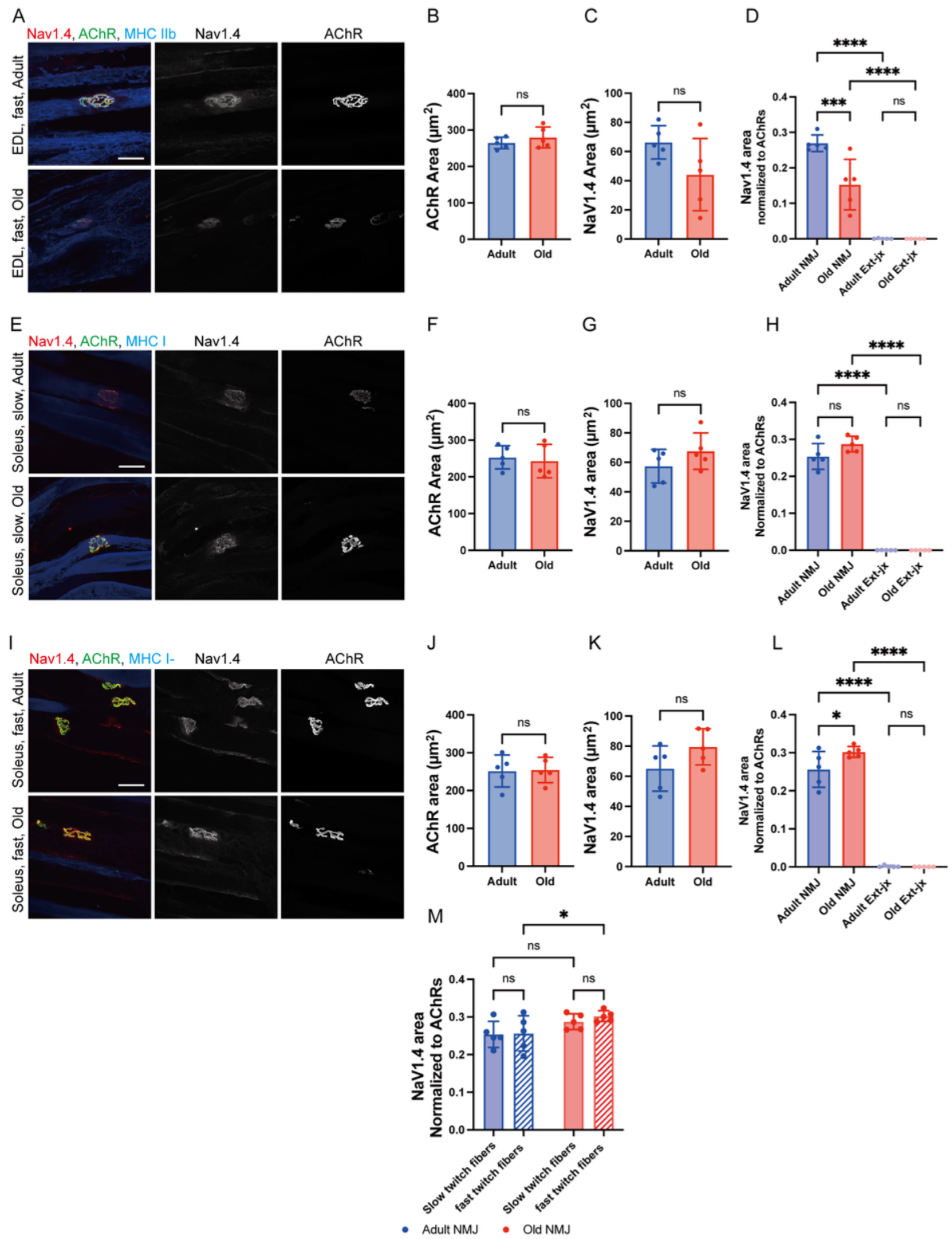

Supplementary Figure 2: Aging alters NaV1.4 expression levels at NMJs in a muscle-dependent manner. NaV1.4 area normalized to NMJ size showed a significant reduction of synaptic NaV1.4 in old extensor digitorum longus (EDL) but not in soleus muscles. Representative confocal images and quantification of NMJs in **(A-D)** EDL muscles, **(E-H)** slow muscle fibers of soleus muscles, and **(I-L)** fast-twitch (MHC I-negative) fibers of soleus muscles from adult (3–4 months) and old (26 months) mice. (**A, E, I.)** Acetylcholine receptors (AChRs, green) were labeled with α-bungarotoxin, voltage-gated sodium channel 1.4 with anti-NaV1.4 (NaV1.4, red), and muscle fibers with anti-myosin heavy chain (**A.** MHC IIb, **E.** MHC I, **I.** MHC I negative, blue) antibodies. Graphs show (**B, F, J**) AChR area, **(C, G, K)** NaV1.4 area at NMJs, **(D, H, L)** NaV1.4 area normalized to AChR area at NMJs or extra-junctional area (Ext-jx). **M.** Comparison of normalized NaV1.4 area at NMJs between fast- and slow-twitch fibers in soleus revealed no fiber-type differences within age groups. Each data point represents an animal (NMJ or extra-junctional). Graphs show mean ± SD. NMJ sampling: 10–14 NMJs analyzed per fiber type in each mouse; n = 5 mice per age group. One-way ordinary ANOVA (junctional vs. extra-junctional), with Sidak post hoc correction, was used for multiple comparisons. ****$p < 0.0001$; $p < 0.05$; ns, not significant. Scale bars: 50 µm.

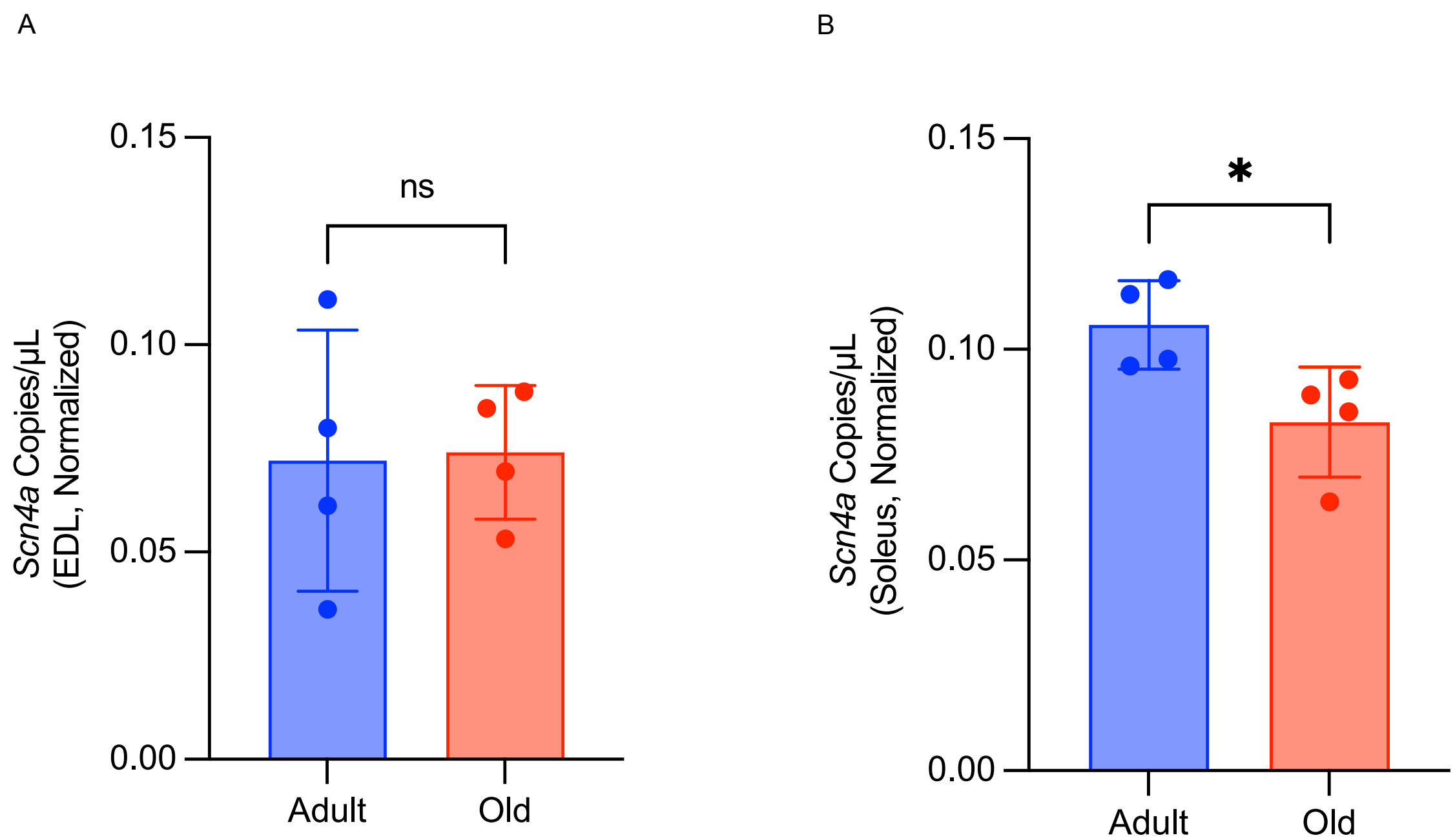

Supplementary Figure 3. Age-related differences in NaV1.4 (Scn4a) mRNA expression in Extensor digitorum longus (EDL) and soleus muscles measured by digital PCR. Absolute *Scn4a* transcript levels were quantified by digital PCR in EDL and soleus muscles from adult (4–5 months, n = 4) and old (27–28 months, n = 4) male C57BL/6 mice. Expression values (copies/µL) were normalized to the average of GAPDH, Rps18, and β-actin. **A.** Scn4a expression in EDL did not differ between age groups. **B.** Soleus muscles from old mice showed significantly lower Scn4a expression compared to adult mice. Data are presented as mean ± SD, and statistical significance was assessed using an unpaired t-test. * $p<0.05$, ns not significant.

A

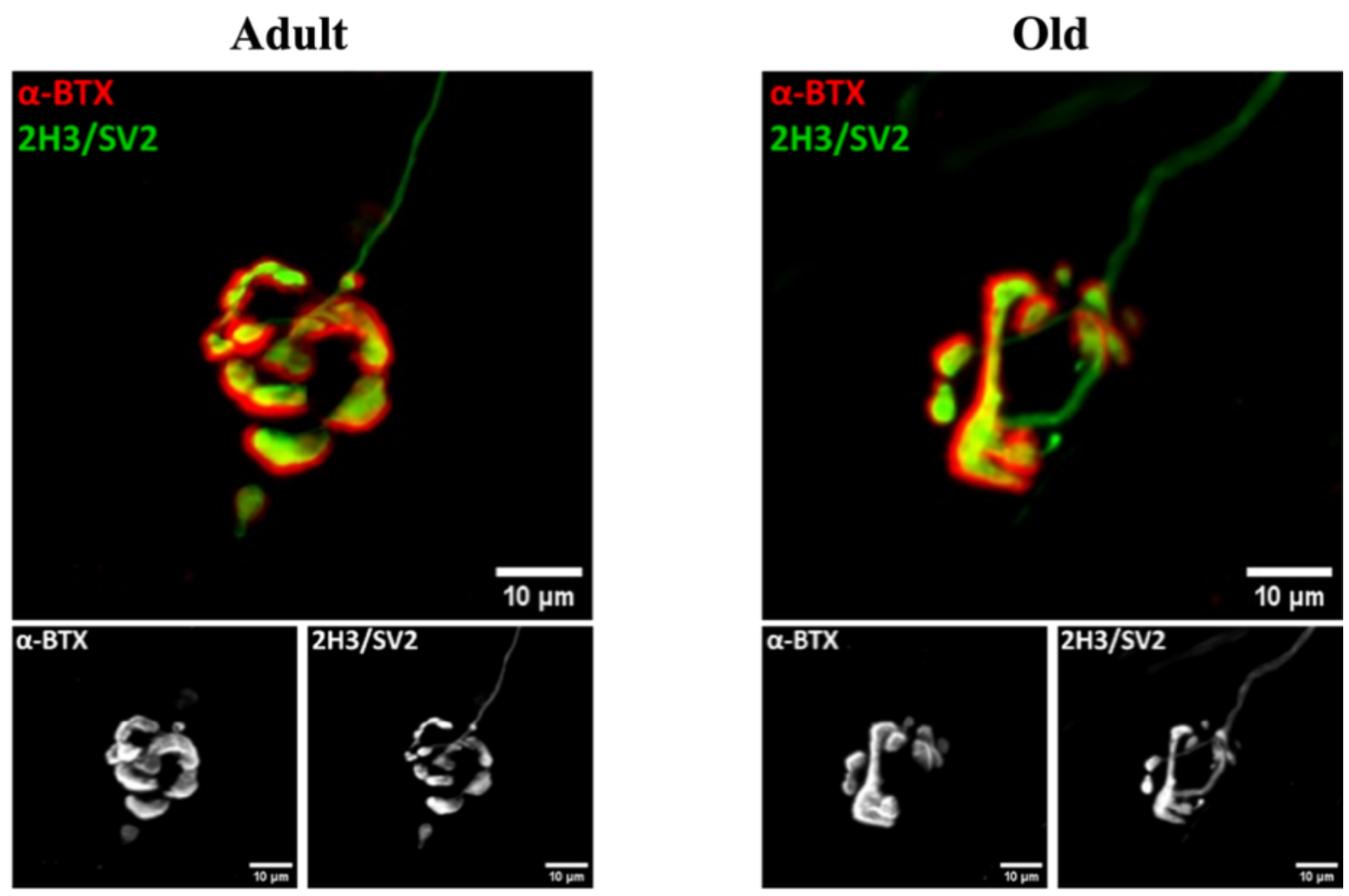

Adult
Old
α-BTX
2H3/SV2
10 µm

B

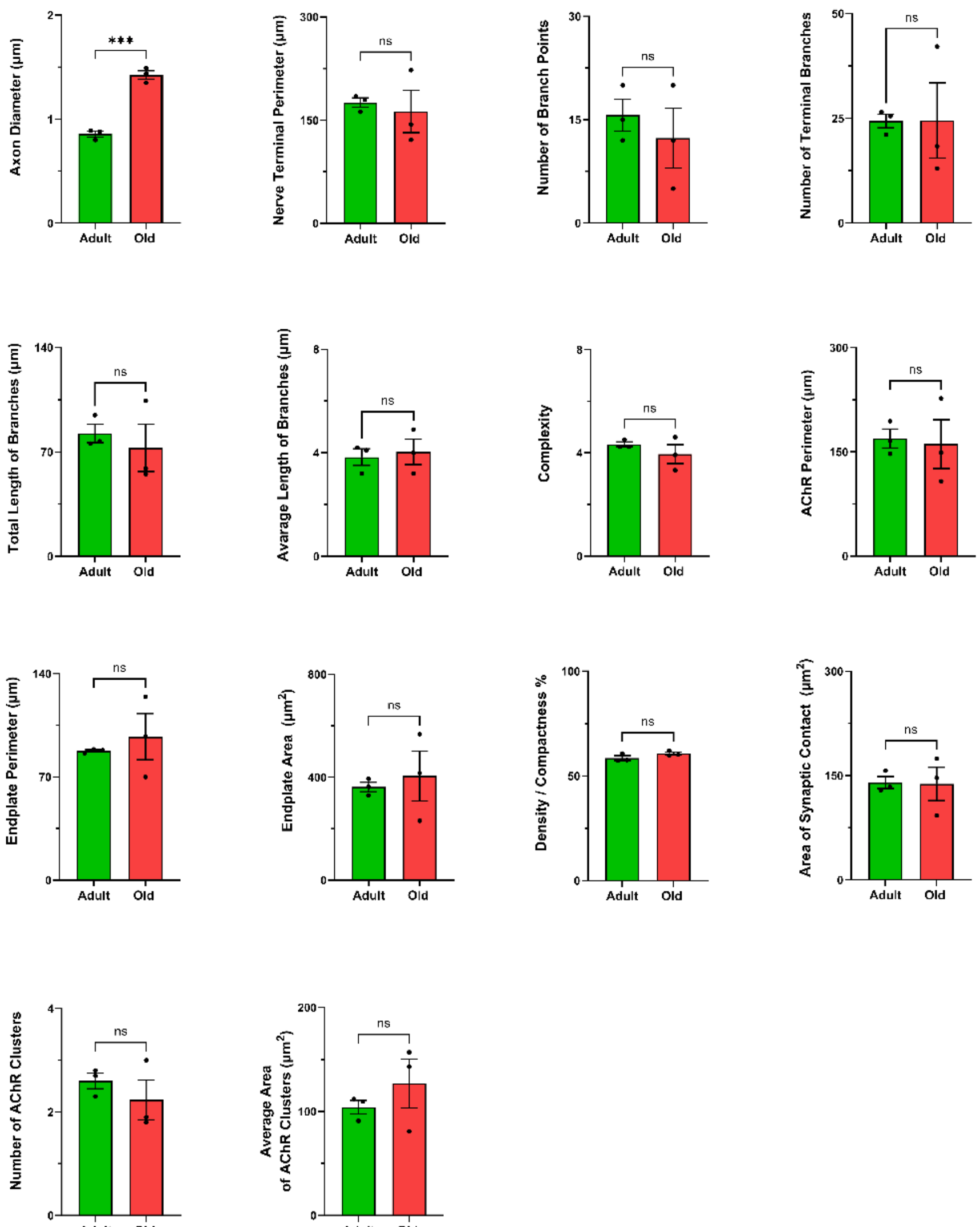

Axon Diameter (µm)
***
Nerve Terminal Perimeter (µm)
Number of Branch Points
Number of Terminal Branches
Total Length of Branches (µm)
Avarage Length of Branches (µm)
Complexity
AChR Perimeter (µm)
Endplate Perimeter (µm)
Endplate Area (µm²)
Density / Compactness %
Area of Synaptic Contact (µm²)
Number of AChR Clusters
Average Area of AChR Clusters (µm²)
ns
Adult
Old

Supplementary Figure 4: Human NMJ morphology and muscle fiber diameter remain unchanged with increasing age in the dorsal interosseous muscle, demonstrating that structural denervation is not a feature of the aging NMJ in humans. **A.** Representative confocal micrographs representing the 'average' morphology of NMJs from the Dorsal Interosseous (DIO) muscle from two distinct age groups (Adult and Old). A-BTX (α-bungarotoxin) has been used to label acetylcholine receptors (red), whilst antibodies against SV2 and 2H3 have been used to label synaptic vesicles and neurofilaments respectively (green). The human NMJ samples shown in panel A are from the same biopsy samples shown in Figure 3A. Scale bar = 10 μm. **B.** Bar charts (mean±SEM) of morphological measurements obtained from human DIO NMJs using NMJ-morph across the two studied age groups. Each data point represents the average of 40 NMJs from one muscle (n = 3 individuals per group). Unpaired t-tests were used for parametric variables, Mann–Whitney tests were used for non-parametric variables. Statistical analyses revealed no significant differences in NMJ morphology between the two age groups across all NMJ morphological variables, except for a significant increase in axon diameter with age. "ns" indicates $p \geq 0.05$; ***$p < 0.001$.

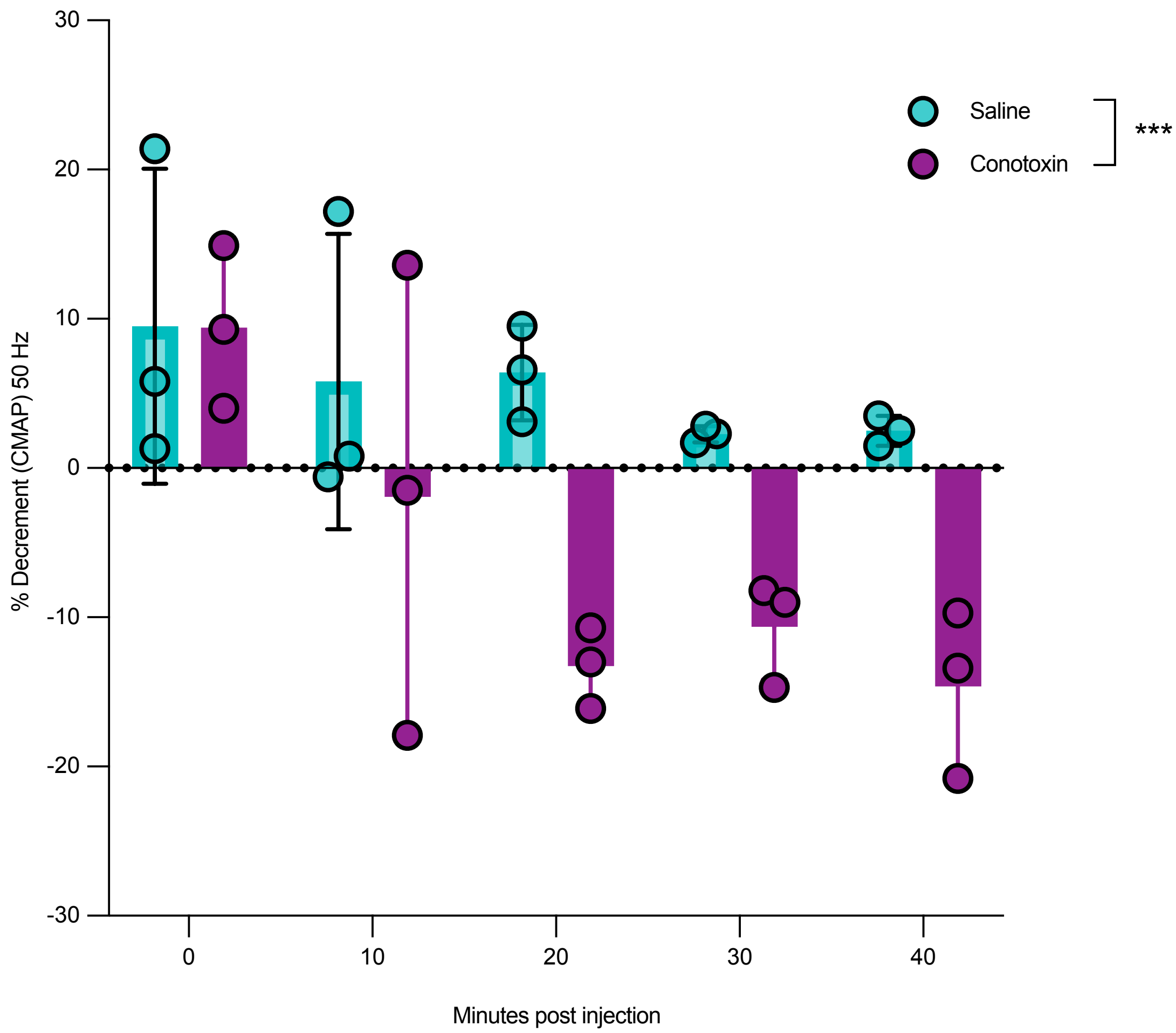

Supplementary Figure 5. μ-Conotoxin impairs NMJ transmission in adult rats. Percentage decrement of compound muscle action potential (CMAP) amplitude during 50 Hz repetitive nerve stimulation (RNS) in adult male Wistar rats (3–4 months old, n = 3 per group) following intramuscular administration of μ-conotoxin GIIIB (purple) or saline (cyan). RNS was performed every 10 minutes for 40 minutes after injection. μ-Conotoxin produced a progressive increase in CMAP decrement beginning ~10 minutes post-injection, whereas saline-injected controls maintained stable responses. Data represent mean ± SD; individual points correspond to animals. Statistical analysis was performed using two-way ANOVA. Two-way ANOVA revealed a significant main effect of treatment (saline versus μ-conotoxin; $F(1,20) = 18.04$, $p = 0.0004$) and a significant main effect of time (minutes following injection; $F(4,20) = 4.31$, $p = 0.0113$) but no significant treatment × time interaction ($F(4,20) = 1.67$, $p = 0.197$).

***Pre-Clinical Studies*: Characterization of Small Molecule Inhibitors of ClC-1**

Two ClC-1 inhibitors (NMD1226 and NMD653) were characterized, however the characterization of only NMD1226 is described here and in **Suppl. Fig. 6** as an example. Characteristics of both NMD1226 and NMD653 are listed in **Suppl. Table 3**.

Confirmation That the Compound Inhibits ClC-1 and That ClC-1 Function Remains Intact in Old Rats. Briefly, the ability of the molecule to inhibit ClC-1 was confirmed using an intracellular microelectrode technique to measure the resting membrane conductance (G_m) of individual muscle fibers in intact muscles from rats. The muscles were dissected out and placed in a perfusing chamber under a microscope, and three microelectrodes were inserted into the same muscle fiber under visual guidance. G_m was measured using a protocol and analysis technique described elsewhere(1). The G_m reflects the function of ion channels that are open at the resting membrane potential and ClC-1 is known to constitute around 80% of this(2). Reduction in G_m after application of a compound is a strong indicator of ClC-1 inhibition but this can be fully confirmed by repeating experiments in Cl^- free solutions in which case any reduction in G_m due to ClC-1 inhibition should be absent. As shown in **Suppl. Fig. 6A** there was concentration dependent reduction in G_m when the molecule was added and this reduction in G_m was absent when experiments were repeated in Cl^- free solution. Finally, when CHO cells expressing the human isoform of ClC-1 were exposed to NMD1226, the ClC-1 current recorded using patch clamping was markedly reduced (**Suppl. Fig. 6B**). In a separate series of measurements, the G_m was found not to be altered in muscles isolated from old rats compared to observations in the 3-month-old rats used in **Suppl. Fig. 6A** (654±8 μS/cm^2 vs 563±37 μS/cm^2 (p=0.0753), respectively). Taken together, these experiments confirmed that NMD1226 inhibits ClC-1 and that ClC-1 function

remains intact in old rats. The latter also aligns with unchanged input resistance and time constant in muscle fibers from old mice.

Confirmation That the ClC-1 Inhibitor Increases Muscle Fiber Excitability. ClC-1 Cl^- ion channel function is a key determinant of skeletal muscle excitability and inhibition of ClC-1 enhances NMJ transmission by increasing the excitability of the muscle fibers. To determine whether the ClC-1 inhibitor would increase muscle fiber excitability, the current required to trigger an AP in muscle fibers was determined using intracellular electrodes (rheobase current). **Suppl. Fig. 6C** shows observations of membrane potential during injections of currents with increasing strength in one muscle fiber before compound addition and in another fiber exposed to high concentration of the compound. The increase of muscle fiber excitability by NMD1226 is evident from the smaller rheobase current and higher number of APs formed at the largest current injections in the fiber at high compound concentration. This experiment was repeated in multiple fibers at different compound concentrations, and as shown in **Suppl. Fig. 6D**, NMD1226 caused a gradual increase in the proportion of muscle fibers that were excited by small amplitude current injections.

Confirmation That the ClC-1 Inhibitor Restores Muscle Function in an Experimental Condition of Compromised NMJ transmission. The increased muscle fiber excitability with NMD1226 prompted an experiment to determine whether NMD1226 could restore muscle function under conditions of compromised NMJ transmission. Nerve-muscle preparations were isolated from adult rats and placed in an experimental setup that enabled measurements of nerve-stimulated force production. Force was first recorded under control conditions and then during exposure to submaximal concentration of an AChR antagonist (tubocurarine). The antagonist imposed NMJ transmission failure as evident from the decline in force production in the

representative traces in **Suppl. Fig. 6E**. When force had declined to a stable level, NMD1226 was added and resulted in notable force recovery confirming that NMD1226 enhanced NMJ transmission and restored muscle function (**Suppl. Fig. 6E**). The experiment was repeated in 6 preparations and from the average observations in **Suppl. Fig. 6F**, it was observed that the ClC-1 inhibitor was able to restore most of the force deficit caused by the transmission failure.

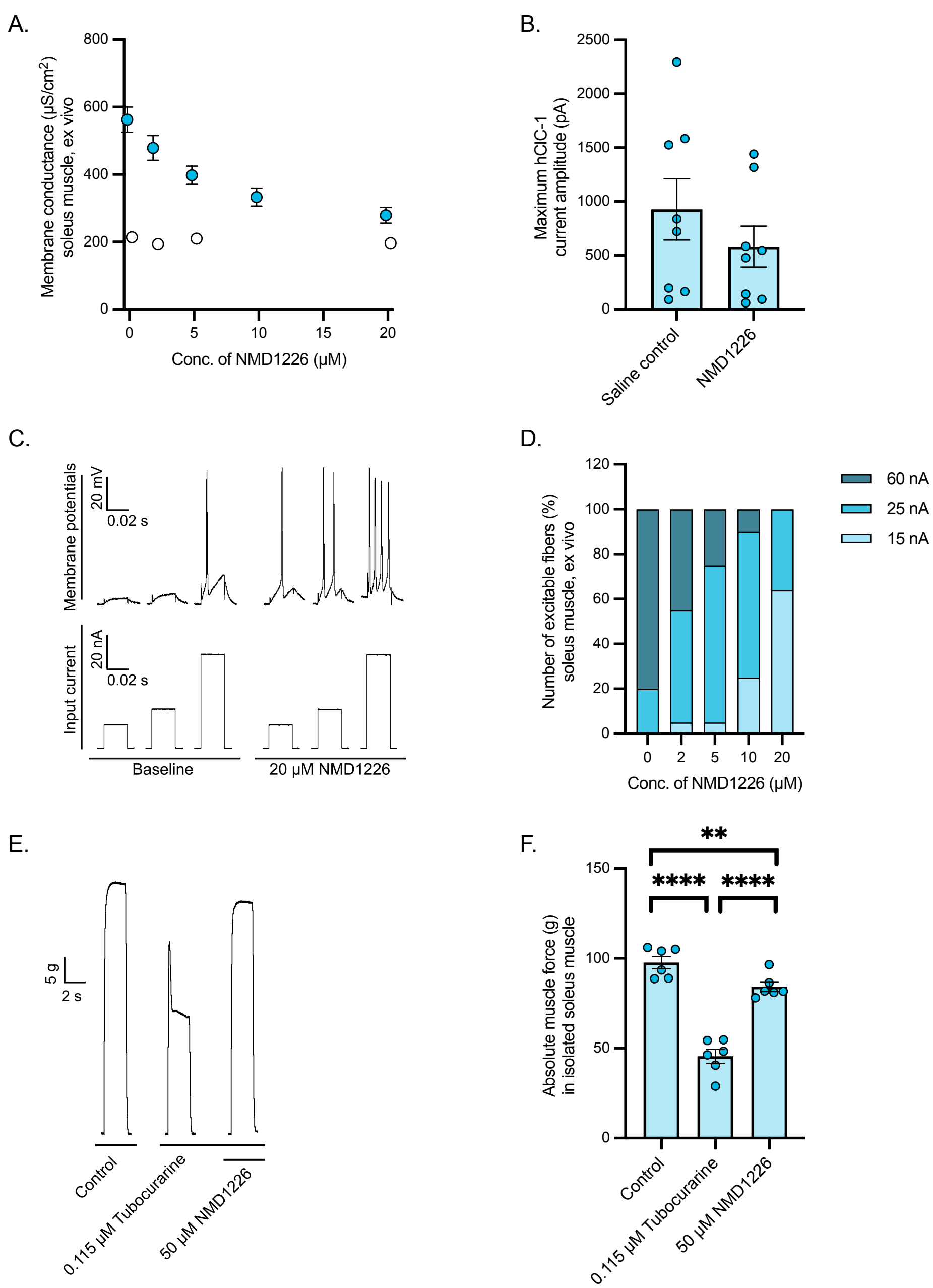

Supplementary Figure 6. Muscle fiber excitability and skeletal muscle force generation are increased in response to ClC-1 channel inhibition. A. Resting membrane ClC-1 conductance (G_m) measured in individual muscle fibers in isolated soleus muscle from 3 month (m) old female rats was decreased by addition of NMD1226 to the experimental chamber in a dose-dependent manner under physiological conditions in normal Krebs-Ringer buffer (blue dots, n=3), but this effect was not apparent in chloride (Cl^-) free solution (white dots, n=1). **B.** Shows average ClC-1 currents in CHO cells expressing the human isoform of ClC-1 channel recorded using an automated patch clamp system before and after addition of the NMD1226. In short, whole cell patches were established and then clamped to a holding potential of -30 mV. Subsequently the voltage was increased to a pre-pulse of +60 mV while recording the maximum ClC-1 activation current. This voltage protocol was repeated while cells were firstly exposed to extracellular (EC) solution with vehicle ("Saline control"). Secondly, cells were exposed to a new portion of EC solution with the small molecule inhibitor, 200 µM ("NMD1226"). The decrease in maximum ClC-1 activation current showed that the small molecule inhibitor was able to block the ClC-1 channel, although not significantly. n=4. **C.** Representative traces illustrating the effect of ClC-1 channel inhibition in a rheobase current test in soleus muscle from 3 m old female rats, *ex vivo*. Each muscle fiber was exposed to an input current of 15, 25, and 60 nA (lower panel) to elicit a membrane potential response in the form of one or more APs (upper panel) reflecting the fiber excitability. Note that increasing concentrations of the ClC-1 inhibitor enhanced excitability of muscle fibers. **D.** Shows distribution of rheobase current in fibers at different concentrations of the molecule. With increasing concentration of the molecule, the fibers became gradually more excitable as shown by the gradual increase in fibers responding to the lower current injections n=2. **E.** Representative traces from experiments where the effect of NMD1226 was evaluated on nerve-stimulated force in isolated soleus muscle from 1 m old male and female rats depressed by tubocurarine. Muscles were stimulated through the nerve every 10 minutes at 60 Hz for 2 s (voltage 12 V, pulse duration 0.02 ms) starting with a control stimulation. Hereafter 0.115 µM tubocurarine was added to the incubation solution and 9 stimulations were performed to allow the effect of tubocurarine to stabilize. Finally, 50 µM NMD1226 was added, and the effect hereof was followed over 6 stimulations. Shown are examples of force before tubocurarine, after equilibrating with tubocurarine and finally when the full effect of the inhibitor had been reached. **F.** Shows summary data from three experimental conditions described in E. Control muscle force was decreased by 53% after addition of tubocurarine, ****$p<0.0001$. The ClC-1 inhibitor was able to restore the force to 86% of the initial force ("Control"), ****$p<0.0001$, but was still lower than the control force, **$p=0.0038$, n=6. P-values reported in F. were derived from a RM one-way ANOVA followed by a Fisher's LSD post-hoc test. Data are mean ± SEM.

Supplementary Table 3. Summary of characteristics of both ClC-1 inhibitors NMD1226 and NMD653.

**Membrane conductance, Isolated soleus muscle fibers**	**NMD1226**	**NMD653**
Average slope<br><br>EC_{50} (µM)	1.8 ± 0.2 (N=3)<br><br>3.7 ± 0.2 (N=3)	2.0 ± 1.1 (N=7)<br><br>4.7 ± 1.9 (N=7)
**Maximum hClC-1 current amplitude, CHO cells**		
Average saline control (pA)<br><br>Average compound (pA)	2295 ± 284.3 (N=4)<br><br>1440 ± 188.9 (N=4)	Not applicable<br><br>Not applicable
**Rheobase current, Isolated soleus muscle fibers**		
Increase of fiber excitability with ClC-1 inhibitor (10 or 12 µM) compared to control (% point)	Current injection of 15 nA: 25 (N=2)	Current injection of 15 nA: 10 (N=2)
**Absolute muscle force, Isolated soleus muscle**		
Average increase at 50 µM ClC-1 inhibitor (% point)	40.1 ± 3.8 (n=6)	29.0 ± 10.2 (n=29)

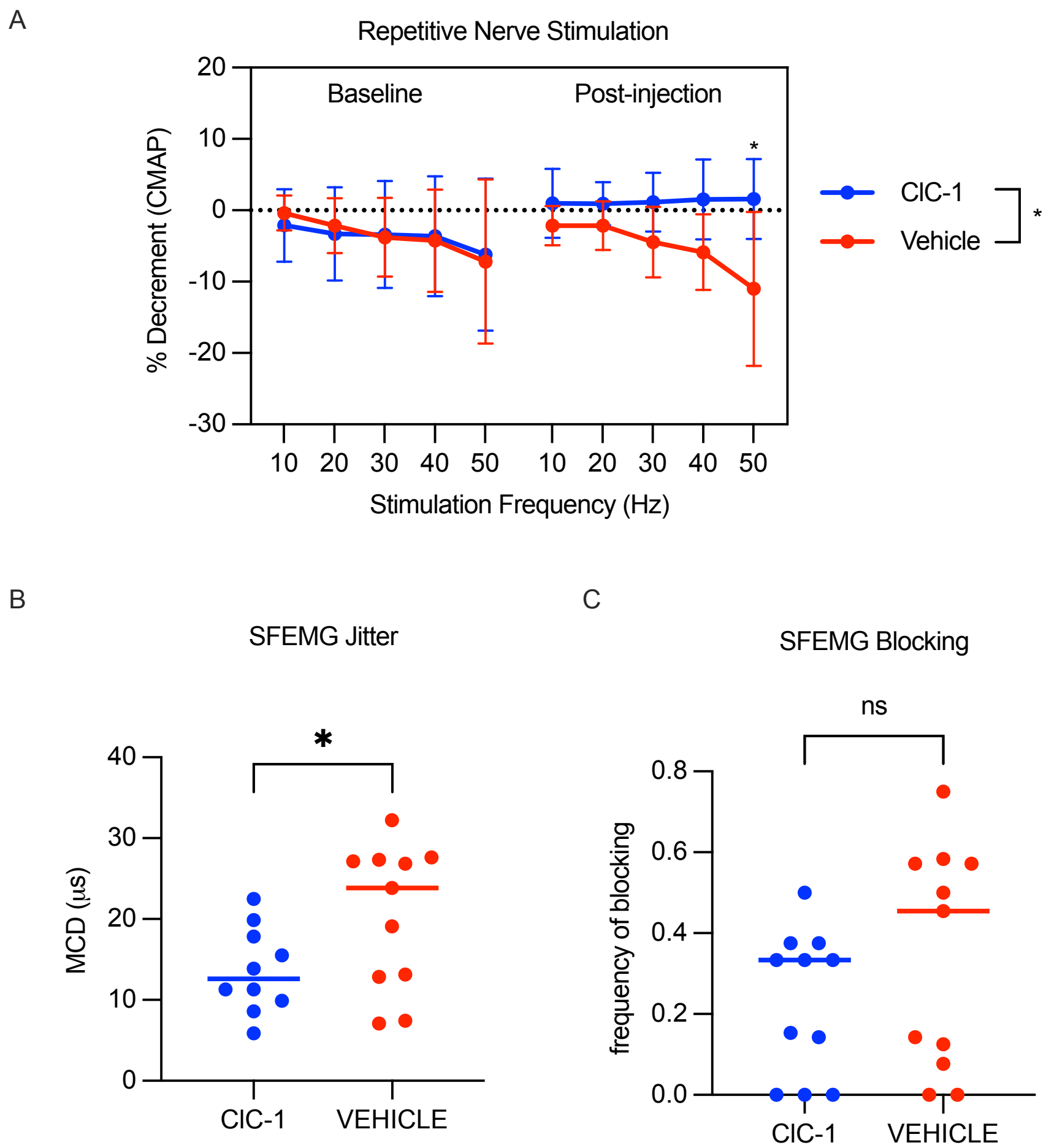

Supplementary Figure 7. Effect of acute ClC-1 inhibition on neuromuscular transmission in aged mice. **A.** Repetitive nerve stimulation (RNS) at baseline and following intraperitoneal injection of the ClC-1 inhibitor NMD653 20 mg/kg [n=11 (6 females, 5 males), mean age: 27.9 month, range 27-29 month] or vehicle [n=11 (4 females, 7 males), mean age: 28.3 months, range age 27-29]. Percent decrement in compound muscle action potential (CMAP) amplitude is shown across stimulation frequencies (10–50 Hz) recorded from the gastrocnemius muscle. A three-way repeated-measures ANOVA showed significant effects of stimulus frequency ($p<0.0001$) and treatment ($p=0.045$), with a significant frequency × treatment interaction ($p=0.0039$). Time and all interactions involving time were not significant, indicating stable frequency-dependent treatment effects across time. Points in graphs represent average decrement across animals. * $p<0.05$ adjusted p values (Tukey's multiple comparisons test) **B.** Single fiber electromyography (SFEMG) jitter quantified as mean consecutive difference (MCD) averaged across recordings from each animal (showed significantly lower jitter in ClC-1–treated mice compared with vehicle ($p=0.0478$, unpaired t-test with Welch's correction), consistent with improved neuromuscular transmission stability. **C.** SFEMG blocking (%) did not differ significantly between groups ($p=0.2746$, unpaired t-test with Welch's correction). Points in SFEMG graphs represent average values for jitter and % of fibers with blocking in each animal. Lines indicate group means. One data point from the ClC-1 group was excluded prior to analysis; no additional outliers were identified using ROUT (Q = 10%) in the remaining dataset *$p<0.05$, ** $p<0.01$

Supplemental Methods

MATERIALS AND METHODS

Clinical Assessment of NMJ Transmission

Study Design. This cross-sectional study (Investigating Loss of Neuromuscular Junction Transmission Fidelity in Older Adults [STAMINA]; NCT04904926) was designed to compare apparently healthy, young, and middle-aged adults (18-50 years) to older adults with clinically meaningful, age-related weakness (70+ years). We sought to balance the groups by sex. To target the selection of weak older adults we recruited older adults in the 80's and 90's who self-reported physical limitations (based on a phone interview). We assessed indices of leg extensor and handgrip muscle strength, physical function, quadriceps muscle size via MRI, muscle strength normalized to muscle volume, and body composition. Additionally, we leveraged stimulated single fiber EMG to assess indices of NMJ transmission in the vastus lateralis.

Muscle Strength & Physical Function. Leg extension maximal voluntary isokinetic strength was measured at 60° per s using a Biodex System 4 Dynamometer (Biodex Medical Systems Inc., Shirley, NY). Participants were seated with their left knee at 90° flexion and the knee axis of rotation in alignment with the rotational axis of the Biodex torque motor. A lap belt was applied to prevent movement at the hip, and the participants' left lower leg was affixed to a lower extremity lever arm, which was attached to the Biodex torque motor. Participants received visual feedback of torque on a monitor located 1 meter in front of them. Six trials were performed with 30 s rest between bouts, and the maximum voluntary isokinetic strength was calculated as the mean of the highest three values of maximal isokinetic torque (Newton-meters, N-m) produced between 90° and 30° of knee flexion. This testing protocol is consistent with the isokinetic leg extension

strength measurement protocol utilized by Manini et al. (2007) when deriving the sex-specific knee extension strength cut points for maintaining mobility (3).

Maximal handgrip strength was assessed in a manner similar to that described by Villanfañe (2015) (4) using a portable JAMAR® Hydraulic Hand Dynamometer; (Model 5030 J1; Lafayette Instrument Co.; Lafayette, Indiana). In short, after demonstrating its use, the participant's hand and fingers were fitted to the dynamometer and subjects performed the test in the upright seated position with the shoulder of the tested arm adducted to the side, the elbow flexed at 90°, and the forearm and wrist aligned in the neutral position. Three trials were performed with 30 s rest between bouts and measurements were recorded to the nearest 0.5 kg. If the top two trials were not within 3 kg, a fourth trial was performed.

We obtained several indices of physical function/mobility. First, a composite short physical performance battery (SPPB) score was calculated based on performance of a series of standing balance tasks, a 5x chair rise test, and a usual 4-meter gait speed test as previously described (5). We also assessed stair climb power and four-square step test time as previously described (6).

Muscle Volume & Body Composition. Quadriceps femoris muscle volume of the left leg was assessed using magnetic resonance imaging (MRI) as previously described (7). In brief, MRI scans were performed with a 0.25-Tesla Musculoskeletal MRI system (Esaote G-Scan Brio, Genoa, Italy) to acquire contiguous transverse T-1 weighted spin echo image slices in the thigh region with a slice thickness of 10 mm and an inter-slice distance of 10 mm. The Medical Image Processing, Analysis, and Visualization (MIPAV) software was subsequently used to calculate quadriceps muscle volume. Fat was subsequently subtracted by applying a shading correction to each image. A total of five slides at the mid-point of the quadriceps was used to calculate muscle

volume with the Cavalieri method. We have previously reported our between-day reliability of the MRI technique to have a coefficient of variation of 1.3% (7).

Percent body fat and appendicular lean mass relative to height^2 was quantified using dual-energy X-ray absorptiometry (DXA) (Hologic Discovery QDR model Series, Waltham, MA, USA). A whole-body scan was performed, and whole-body and regional body composition measurements were determined using the system's software package (Hologic APEX, Version 4.0.2). Subjects were advised to report to the laboratory in a hydrated state and they were given scrubs to wear during the scan. They were also given the opportunity to use the restroom prior to the scan. Care was taken to follow The International Society for Clinical Densitometry guidelines for positioning during the scan (8). We have previously reported our between-day reliability of the DXA technique to have a coefficient of variation of 0.96% for estimating lean mass (7).

Clinical Stimulated Single Fiber EMG. We assessed NMJ transmission with stimulated SFEMG using a Sierra Summit clinical electrodiagnostic system (Cadwell, Kennewick, WA, USA) (9). First, the region of the motor point of the left vastus lateralis muscle was localized by eliciting muscle twitches with the minimal possible surface stimulation (Stimulation: duration: 0.1-0.2 ms, current intensity: 0-50 mA). Then, two insulated 28-gauge monopolar needle stimulation electrodes (Natus Neurology, Middleton, WI, USA) were inserted at or just proximal to the motor point localization. A highly selective SFEMG recording electrode (25 x 0.30 mm, SEI EMG s.r.l., Cittadella, IT) was then inserted ~2-3 cm distal to the stimulating electrodes. Terminal twigs of the femoral nerve were stimulated at 10 Hz while adjusting both the stimulation parameters (duration: 0.05-0.1 ms, intensity: 0-5 mA) and the positions of the electrodes (stimulating and recording) as needed to isolate single muscle fiber APs.

Standard definitions of suitable SFEMG single muscle fiber APs were used such that each single muscle fiber AP that was included met the following criteria: a stable, all-or-none response without fractionation, a rise time of <300 μs, and a minimum amplitude of 200 μV (10). Once one or more suitable single muscle fiber APs were localized, stimulation intensity was adjusted to ensure supramaximal axonal stimulation. Then, a total of 50-100 consecutive stimulations (at 10 Hz) were analyzed at each synapse for NMJ blocking (i.e. NMJ transmission failure) and jitter (i.e. NMJ transmission variability). Responses with jitter values less than 5 μs were not accepted in order to exclude jitter recordings from possible split muscle fibers or direct muscle stimulation. To adequately sample the muscle, 3-4 skin insertions were performed to samples different areas.

Muscle Fiber and NMJ Excitability in Rodents

Isolated Single Muscle Fiber Excitability in Aged Mice. Adult (6-7 months, 5 males, 5 females) and old (26-27 months, 5 males, 4 females) C57BL/6N mice (National Institute of Aging, aged rodent colonies) were sacrificed using CO_2 inhalation followed by cervical dislocation. The extensor digitorium longus (EDL) muscle was dissected out intact. Muscles were maintained and recorded from within 6 hours of sacrifice. The recording chamber was continuously perfused with Ringer solution containing (in mM): NaCl, 118; KCl, 3.5; $CaCl_2$, 1.5; $MgSO_4$, 0.7; $NaHCO_3$, 26.2; NaH_2PO_4, 1.7; glucose, 5.5 (20-22°C) and equilibrated with 95% O_2 and 5% CO_2. Contraction was prevented by loading muscles with 50μM BTS dissolved in DMSO for 45 minutes prior to recording. Following pinning, muscle strips were stained with 10 uM 4-Di-2ASP and imaged with an upright epifluorescence microscope. At this concentration, 4-Di-2ASP staining enabled visualization of surface nerve terminals as well as individual surface muscle fibers. The fibers were impaled with 2 electrodes with resistances between 10 and 30 MΩ. The first electrode was used for current injection and the second to record membrane potential. Electrodes were filled with 3

M KCl solution containing 1 mM sulforhodamine 101 to allow for visualization. Capacitance compensation was optimized prior to recording. Action potentials were evoked by a 0.2 ms injection of current ranging from 100 to 1000 nA. Membrane time constant and input resistance were measured during a 200 ms injection of hyperpolarizing current ranging from 5 to 40 nA. Fibers with resting potentials more depolarized than –74 mV were discarded. Sampling frequency was 50 kHz with a 5 kHz low pass filter.

Junctional and Extra-junctional Excitability in Aged Mice. For studies of excitability near (junctional) and far from the NMJ (extra-junctional), EDL muscle fibers were imaged and impaled with a pair of sharp electrodes both within 100 µm of the NMJ (junctional) and greater than 1 mm from the NMJ (extra-junctionally). Half the fibers were initially impaled near the NMJ and half were initially impaled away from the NMJ to control for loss of resting potential with repeated impalements. Re-impalement caused loss of 10 to 20 mV of resting potential by the end of the recordings, but we found that this degree of depolarization did not alter the amount of current necessary to trigger an AP. The amount of current injected was 1000 nA and the duration of the current injection was varied between 0.2 and 1 ms. The integral of current and duration of stimulation was used to determine the amount of charge required to trigger an action potential near versus far from the NMJ. To control for potential impact on excitability of the resting membrane potential changing during recordings, the order of impaling and recording was varied across fibers (junctional versus extra-junctional).

Junctional and Extra-junctional Excitability in Aged Rats. For studies of excitability in rats, male Wistar rats, adult (6-7 months old, n=5) and old (23-24 months old, n=7) were euthanized and EDL muscles were dissected and prepared for recording. The protocol involved 3 sharp electrodes: two recording (E1 and E2) and a stimulation electrode (ES). E1 was inserted into a

muscle fiber at the NMJ, while E2 was inserted at least 800 μm away from E1 to perform extra-junctional recordings. Proximity to NMJ was verified by the presence of miniature endplate potentials. ES was first inserted close to the E1 (distance < 50 μm) to inject current at the NMJ, before being removed and reinserted near E3 to repeat the stimulation protocol. The stimulation protocol involved injecting square currents lasting 20 ms every 500 ms. The amplitude of the current injection started at 10 nA and was increased by 2 nA every subsequent current injection. When the current amplitude was sufficiently large to elicit an AP, the recordings stopped and the AP inducing current was taken as the excitation current threshold. The order of the recording site was alternated between every fiber (e.g. 1st fiber: junctional followed by extra-junctional, 2nd fiber: extra-juntional followed by junctional). Excitation of muscle fiber followed by re-impalement by ES would occasionally cause the resting potential to shift; if the resting potential was changed by 10mV or more, the recording was discarded.

Junctional and extra-junctional recordings were analyzed to assess localized postsynaptic excitability ("gain"), defined as requiring less stimulation to trigger an AP at the NMJ than in extra-junctional regions. Each fiber was classified dichotomously as either exhibiting gain or not, with gain present when the junctional AP current threshold was lower than the extra-junctional threshold (junctional-to-extra-junctional rheobase ratio <1.0). Using a binary measure aligns with the all-or-none nature of NMJ transmission and provides a statistically robust metric at the single NMJ level, allowing direct comparison of the proportion of fibers that retain the gain to facilitate reliable NMJ activation. To control for potential impact on excitability of the resting membrane potential changing during recordings, the order of impaling and recording was varied across fibers (junctional versus extra-junctional).

Pre-Clinical and Clinical Assessment of NaV1.4 Channel Density at the NMJ

Mouse NMJ sample preparation, staining and imaging. ***Sternomastoid muscle dissection and preparation:*** Adult (3-4 months, n= 4) and old (26-28 months, n= 4) C57BL/6N mice (National Institute of Aging, aged rodent colonies) were euthanized using isoflurane inhalation, followed by cervical dislocation to preserve neck muscle integrity. The sternomastoid muscles were dissected following exposure of the neck region, pinned in a Sylgard dish, and isolated using forceps. Muscles were then cleaned of connective tissue using fine forceps under a dissecting microscope, rinsed in phosphate-buffered saline (PBS), and fixed in 4% paraformaldehyde (PFA) for 20 minutes. Following fixation, muscles were filleted longitudinally into four pieces and washed three times with phosphate-buffered saline (PBS). ***Whole-mount muscle staining:*** For immunofluorescence staining, muscles were permeabilized in 2% Triton X-100 in PBS for 30 minutes. Blocking was then performed using a buffer containing 10% normal goat serum and 1% Triton X-100 in PBS for another 30 minutes. Primary antibodies, including mouse monoclonal anti-NaV1.4 (1:1000, Antibodies Inc N255/38) to visualize NaV1.4, were diluted in the blocking buffer and incubated overnight at 4°C. Following this, muscles were washed four times for 20 minutes each in 1% Triton X-100 in PBS. The secondary antibody (1:200, AlexaFluor 594 goat anti-mouse IgG2a, Invitrogen, A-21135) and AlexaFluor 488-conjugated α-bungarotoxin (1:40, Invitrogen B13422) for AChR labeling were applied for 2 hours at room temperature. Muscles were washed again four times for 20 minutes each in 1% Triton X-100 in PBS. Then, the muscle tissue was teased apart with forceps and mounted on clean slides. ***Confocal imaging:*** Confocal images were acquired using a 63X/1.4 oil immersion objective Leica TCP SP8 microscope. Z-stack images were captured at 0.5 μm intervals with a 2× zoom and a frame size of 1024 × 1024

pixels. The green channel (α-bungarotoxin) was excited at 488 nm, and the red channel (NaV1.4) at 594 nm. For each sample (n=8; 4 adult, 3-4 months old and 4 old mice, 26-28 months old), 24-27 NMJs were imaged, resulting in a total of approximately 207 NMJs analyzed. ***Image analysis:*** NaV1.4 and AChR areas were quantified using Fiji/ImageJ software. Confocal Z-stack images were first processed to generate maximum intensity projections, and the color channels were split to isolate AChR and NaV1.4 signals. The region of interest (ROI) was defined by manually tracing the AChR signal, expanding the selection by 2 µm to include NaV1.4 labeling. The image scale was set using known pixel dimensions, and appropriate thresholds were applied to outline the areas of interest. The NaV1.4 area was calculated relative to the AChR signal, which we used to define the ROI for each NMJ. Specifically, the NaV1.4 area was quantified within the same ROI outlined by the AChR signal, ensuring that NaV1.4 measurements were normalized to the size of the individual NMJ. This normalization accounts for variability in NMJ size between samples and groups. All measurements were initially recorded in pixels and subsequently converted to μm^2 using the established calibration scale for accurate quantification.

Mouse EDL and soleus muscles dissection, preparation, and imaging. EDL and soleus muscles were dissected from different cohorts of mice to examine NaV1.4 expression in muscles with distinct fiber-type composition. Muscles were fixed in 1% PFA for 20 minutes at 4°C and rinsed thoroughly in PBS. The muscles were then incubated in 20% sucrose at 4 °C overnight for cryoprotection. Then embedded in OCT, frozen in isopentane cooled with liquid nitrogen, and cryosectioned longitudinally at 20 µm thickness using a Leica cryostat maintained at –20°C. Sections were mounted on glass slides and air-dried. Sections were surrounded with a hydrophobic barrier (PAP pen) and blocked for 1 hour at room temperature in PBS containing 10% normal goat serum and 1% Triton X-100. Primary antibodies were diluted in the same blocking buffer and

incubated overnight at room temperature. The following primary antibodies were used: mouse anti-NaV1.4 (1:1000, clone N255/38, IgG2a, Antibodies Inc.), mouse anti–myosin heavy chain (MHC) type I (1:1000, clone A4 951, IgG1, Developmental Studies Hybridoma Bank), and mouse anti-MHC type IIb (1:300, clone BF-F3, IgM, Developmental Studies Hybridoma Bank). After four 5-minute washes in PBS, sections were incubated for 2.5 hours at room temperature with secondary antibodies and α-bungarotoxin–Alexa Fluor 488 (1:1000; Invitrogen, B13422) to label AChRs. Secondary antibodies included goat anti-mouse IgG1–Alexa Fluor 647 (1:500; Invitrogen A21240), goat anti-mouse IgM–Alexa Fluor 647 (1:500; Invitrogen, A21238), and goat anti-mouse IgG2A–Alexa Fluor 594 (1:500; Invitrogen, A21135). After staining, slides were washed four times in PBS, briefly rinsed with Milli-Q water, and mounted with Immu-Mount (Epredia, 9990402). ***Confocal imaging:*** Confocal images were acquired using an Olympus confocal microscope equipped with a 63×/1.4 NA oil-immersion objective. Z-stack images were collected at 0.45 µm intervals with a 2× digital zoom and a frame size of 1024 × 1024 pixels. The green channel (α-bungarotoxin) was excited at 488 nm, the red channel (NaV1.4) at 594 nm, and the far-red channel was used to detect myosin heavy chain type I or IIb to distinguish slow- and fast-twitch fibers in soleus and EDL, respectively. For each sample (n = 10; 5 adults aged 3–4 months and 5 old mice aged 26–28 months), 10–14 NMJs were imaged per muscle, resulting in approximately 50-55 NMJs analyzed in each group. **Image analysis:** Quantification of NaV1.4 signal intensity was performed in Fiji/ImageJ (NIH) using custom-made macros for batch processing on maximum-intensity projections of confocal Z-stacks acquired under identical imaging parameters. The junctional ROI corresponding to the AChR-positive NMJ was manually defined using the α-bungarotoxin (BTX) signal. To define the extra-junctional ROI, the same BTX-based junctional ROI was manually dragged at least 20 µm away from the BTX signal along

the same muscle fiber, ensuring that NaV1.4 signal was measured within the same fiber but outside the synaptic region. Both ROIs were saved following a standardized naming format and analyzed in batch using these custom macros. For each image, NaV1.4 area and mean signal intensity were measured before and after applying a threshold. An automated Otsu threshold was first used to determine threshold ranges across all samples; the averaged minimum and maximum threshold values were then applied as a fixed threshold to ensure consistent quantification across images. The NaV1.4 area was normalized to the BTX-defined area of the same ROI. Data were exported as CSV files, compiled in Excel, and analyzed in GraphPad Prism for group comparisons of normalized NaV1.4 area and intensity.

Rat NMJ sample preparation, staining and imaging. Male Wistar rats (RjHan:WI, Janvier Labs, FR) were either adult (mean age: 6.4±0.37 months; range: 5.5-7 months, "Adult rats") or aged (mean age: 21.8±0.49 months; range: 21-23 months, "Old rats"). They were euthanized and lumbrical muscles dissected according to Sleigh et al. 2014 (11). Lumbrical muscles from the left hindlimb foot were fixated 15 min on ice, washed with PBS, and kept in PBS at 4°C overnight. The following day, further connective tissue was removed from the two most lateral lumbricals from each animal and the fibers slightly teased by spreading out the muscle with a forceps. Muscles were permeabilized with 2% Triton-X100 in PBS for 30 min and blocked with 4% BSA/1% Triton/PBS for 30 min, before incubation overnight at 4°C with primary antibodies (rabbit-anti-scn4a, Alomone Labs, ASC-020, 1:500. Next day, muscles were washed 4x in PBS for a total of 1.5 h and incubated 2 h with secondary antibodies and α-BTX at RT (1.5 µg/mL α-Bungarotoxin-555, Invitrogen, B35451 + 4 µg/mL donkey-anti-rabbit-488, Invitrogen, A32790). They were washed 3x 20 min in PBS, 10 min in PBS + Hoechst 1.54 µg/mL, and 10 min in PBS, before being

mounted with Dako fluorescent anti-fading mounting medium and vaseline in corners of cover glass on objective slides. A Zeiss LSM800 laser scanning confocal microscope with a 40X oil objective was used to acquire z-stack projections of individual en-face NMJs. Images were analyzed using ImageJ software and 23-26 NMJs were analyzed from each animal with n=5 in each group. Endplates were manually traced and the area of NaV1.4 measured.

Human NMJ sample preparation, staining and imaging. Small blocks of tissue, containing full-length muscle fibers from origin to insertion (approx. 2cm in length) were removed from each of the muscles selected and immediately fixed in 4% paraformaldehyde for 1 hour. All specimens were then transferred from theatre to laboratory for immediate processing. Following muscle harvest, small bundles of 25-30 muscle fibers were teased out from the larger blocks, and of a size suitable for whole-mount preparation. NMJs were then immunolabelled for presynaptic 3A10/SV2 proteins or Nav1.4, and postsynaptic AChRs according to the following protocol (antibodies and concentrations are listed below): α-bungarotoxin for 30 minutes; 4% Triton X for 90 minutes; 'block' for 30 minutes (4% bovine serum albumin and 2% Triton X); primary antibodies (in block) for 3 nights at 4°C; 4 washes of 1xPBS; secondary antibodies for 1 night at 4°C; 4 washes of 1xPBS. Preparations were then whole-mounted in Mowiol and stored at -20°C prior to imaging. Primary antibodies: anti-Neurofilament-associated mouse IgG1 (1:50 dilution, Developmental Studies Hybridoma Bank, 3A10, AB_531874), anti-SV2 mouse IgG1 (1:50 dilution, Developmental Studies Hybridoma Bank, SV2, AB_2315387), anti-Nav1.4 Sodium Channel mouse IgG2a (1:1000 dilution, Antibodies Incorporated, N255/38). Secondary antibodies: Alexa Fluor 488 donkey anti-mouse IgG (1:400 dilution, Thermo Fisher Scientific). TRITC conjugated α- bungarotoxin (1:500 dilution, BTIU00012, VWR International Ltd).

Images were acquired and analyzed using a standardized workflow, 'NMJ-morph', as previously described (12). Briefly, a Zeiss LSM 710 confocal microscope was used to acquire z-stack projections of individual en-face NMJs, including a short length of their pre-terminal axon. Images were then analyzed according to the described workflow, which uses ImageJ or Fiji (and freely availably plugins) to measure 21 individual pre- and post-synaptic morphological variables. Again, based on the NMJ-morph recommendations, 40 NMJs per muscle were analyzed to achieve accuracy of reported mean values. Following confocal microscopy, the teased preparations were re-imaged at x20 magnification using an Olympus IX71 microscope, Hamamatsu C4742-95 camera and Openlab Improvision software. Measurement of muscle fiber diameter was performed manually in ImageJ; again, 40 individual fibers were analyzed per muscle.

To measure parajunctional NaV1.4 rim width, confocal micrographs (z stack maximum intensity projection of both NaV1.4 and BTX signal) of individual, *en face* NMJs were analyzed using ImageJ. Mean rim width was calculated by averaging 3 independent manual measurements of rim width (distance extending from the edge of the AChR label to the outer edge of the NaV1.4 signal), attempting to incorporate measurements of widest and narrowest regions of the NaV1.4 rim. For comparative analysis of NaV1.4 area between adult and old human NMJs, a nearest-neighbor matching algorithm was applied to pair NMJs based on similarity in AChR area. Each old NMJ was matched to the adult NMJ with the most comparable AChR area by minimizing absolute differences across all possible pairs. Once a match was established, the corresponding adult NMJ was removed from subsequent pairings to maintain one-to-one matching. Pairs differing by more than 10% in AChR area were excluded, resulting in 34 matched NMJ pairs included in the final quantitative analysis. Because each older NMJ was explicitly matched 1:1 to an adult NMJ based on AChR area ($\leq$10% difference) and the resulting dataset consisted of dependent

paired observations rather than independent samples, within-pair differences were therefore analyzed using a paired t-test (13, 14).

Digital PCR (mRNA Expression). Soleus and EDL muscles were collected from adult (4–5 months, n = 4) and old (27–28 months, n = 4) male C57BL/6 mice. Mice were deeply anesthetized and euthanized by cervical dislocation. Muscles were rapidly dissected, wrapped in aluminum foil, flash frozen in liquid nitrogen, and stored at –80°C until processing.

Total RNA was isolated using TRIzol reagent (Thermo Fisher Scientific, Waltham, MA, USA). Approximately 400 µL of TRIzol and 80 µL of chloroform were added to each sample along with stainless-steel homogenizing beads, and tissues were homogenized and centrifuged at 12,000 × g to separate the phases. The aqueous phase was transferred to a clean tube and purified using the Quick-RNA MicroPrep Kit (Zymo Research, Irvine, CA, USA) according to the manufacturer's instructions. From the purified RNA, 800 ng was used for cDNA synthesis with the SuperScript IV VILO Master Mix (Thermo Fisher Scientific) in a 20 µL reaction volume. The resulting cDNA was diluted 1:20 with nuclease-free water prior to use in digital PCR (dPCR) reactions and stored at –20°C until analysis. Digital PCR was performed on a QuantStudio Absolute Q Digital PCR System (Thermo Fisher Scientific) using pre-designed and pre-validated TaqMan assays. The assay ID for NaV1.4 (Scn4a) was Mm00500103_m1. Multiplex assays were custom-validated by the manufacturer to prevent probe cross-reactivity. Each 10 µL dPCR reaction contained 2 µL of 1× Absolute Q DNA Digital PCR Master Mix, 0.5 µL of each primer/probe mix (0.5× final concentration), 1 µL of diluted cDNA template, and nuclease-free water to a final volume of 10 µL. Reactions were loaded onto a MAP16 plate (Thermo Fisher Scientific) and run with the following thermal cycling conditions: preheating at 96°C for 10 minutes, followed by 40 cycles of

denaturation at 96°C for 5 seconds and annealing/extension at 60°C for 15 seconds. Following amplification, absolute transcript copy numbers (copies/µL) were quantified using QuantStudio Absolute Q Software, version 6.3. Expression of NaV1.4 was normalized to the average copy number of three housekeeping genes: GAPDH (Mm99999915_g1), Rps18 (Mm02601777_g1), and β-actin (Mm02619580_g1) for each sample.

SFEMG recording following µ-Conotoxin Administration. Experiments were conducted on male Wistar rats (Charles River Laboratories, 3-4 months old, n = 3 per group). Animals were anesthetized with isoflurane and placed on a thermostatically controlled platform to maintain body temperature at 37°C during experiments. µ-Conotoxin GIIIB (Alomone Labs, Jerusalem, Israel) was administered intramuscularly into the gastrocnemius muscle at a dose of 166 ng/g body weight in a total volume of 200 µL, delivered as four separate 50 µL injections distributed across the mid-belly of the muscle near NMJ-rich regions. Control animals received an equivalent volume of sterile saline. Injections were performed in one hind limb, and the contralateral limb was used for recording electrophysiological responses as µ-Conotoxin GIIIB distributes systemically and a more suitable blocking level was obtained in the contralateral limb.

Following µ-Conotoxin GIIIB or vehicle injection for model induction, *in vivo* electrophysiological assessments, including compound muscle action potential (CMAP) recordings and repetitive nerve stimulation (RNS), were performed by an unblinded investigator to evaluate neuromuscular transmission, as described previously (Padilla et al., 2021). A train of 10 supramaximal stimuli was delivered to the sciatic nerve at 50 Hz, and CMAP amplitudes were recorded for each response in the train. RNS trains were repeated at defined intervals (every 10 minutes for a total duration of 40 minutes) to assess changes in transmission over time.

Neuromuscular transmission failure was quantified as the percentage decrement between the first and tenth CMAP responses using the following equation:

$$\% \textbf{ CMAP Decrement} = \frac{\textbf{Amplitude}_{10} - \textbf{Amplitude}_{1}}{\textbf{Amplitude}_{1}} \times \mathbf{100}.$$

More negative values reflect greater impairment of synaptic transmission during repetitive activation. In saline-injected controls, CMAP amplitudes remained stable throughout the experiment, whereas μ-conotoxin treatment produced a progressive 10–20% decrement in CMAP amplitude beginning approximately 10–20 minutes post-injection, indicating impaired neuromuscular transmission. SFEMG recordings were subsequently performed 40 minutes post-injection by a blinded investigator. SFEMG was performed by a blinded investigator in a manner similar to the aforementioned human studies, to quantify jitter and blocking of the gastrocnemius muscle (10). To understand the rate dependent change of jitter and blocking, SFEMG recordings were obtained at 5, 10, and 20 Hz stimulation rates. Jitter (mean consecutive delay) and blocking were evaluated from 50-100 stimulations per synapse and compared between vehicle versus μ-conotoxin GIIIB injected rats.

Investigation of ClC-1 Inhibition to Enhance NMJ Transmission and Muscle Function

Animals used for characterizing the small molecule as a ClC-1 inhibitor: Two age groups of Wistar rats (RjHan:WI, Janvier Labs, FR) were used in an established screening cascade examining ClC-1 inhibitors: One-month-old rats of mixed sexes (50/50) were used for experiments in which nerve-stimulated force was recorded in isolated nerve-muscle preparations (see below). Furthermore, muscles from 3-month-old female rats were used for measurements of membrane conductance (G_m) and rheobase current (see below).

Adult and aged rats for testing the ClC-1 inhibitor: Male Wistar rats (RjHan:WI, Janvier Labs, FR) were either adult (mean age: 6.8±0.28 months; range: 6-7 months, "Adult rats") or aged (mean age: 22.2±0.63 months; range: 19-24 months, "Old rats"). The rats were housed for months and were observed and weighed once per week. At appropriate times, rats were examined using SFEMG technique (see below). Later, the same rats were included in a terminal experiment in which the rats were anesthetized, and nerve-stimulated force was measured as described below.

A group of 20-month-old male Wistar rats were reserved for a 7-day blinded dosing study, which is described below. During the study, the animals were weighed every morning, and the well-being of the rats was checked.

Membrane conductance and rheobase in isolated muscle fibers. G_m in single muscle fibers was measured to confirm that the designed small molecule inhibitors NMD1226 and NMD653 inhibits the ClC-1 channel. Soleus muscles were dissected from 3-month-old rats and the muscles were mounted in a chamber perfused with Normal Krebs-Ringer (NKR) solution containing (mM): 122 NaCl, 25 $NaHCO_3$, 2.8 KCl, 1.2 KH_2PO_4, 1.2 $MgSO_4$, 1.3 $CaCl_2$, 5.0 D-glucose. The Cl^- free NKR solution contained (mM): 122 CH_3NaO_4S, 25 $NaHCO_3$, 2.8 CH_3KO_4S, 1.2 KH_2PO_4, 1.2 $MgSO_4$, 1.3 $CaNO_3$, 5.0 D-Glucose. The perfusion solution was continuously equilibrated with a mixture of 95% O_2 and 5% CO_2, pH ~7.4, at a temperature of 30-31 °C. Muscles were allowed to rest for 30 minutes in the solution before beginning the experiment.

The electrophysiological setups that were used to measure G_m has been described in detail elsewhere (1). Briefly, 3 microelectrodes were inserted into the same muscle fiber under visual guidance. All 3 electrodes recorded the membrane potential, and 2 electrodes were used to inject current. The current injection protocol and the mathematical analysis can be found elsewhere (1, 15).

After determination of G_m was completed in a muscle fiber, the rheobase current, i.e., the required current to elicit an AP in said muscle fiber, was next determined. This was done to investigate fiber excitability before and after addition of NMD1226 or NMD653. To do this, the fiber was exposed to a series of 25 ms positive current injections through one electrode being 15, 25, and 60 nA.

In each experiment, G_m and rheobase current were determined in the control situation ("Baseline") in a range of muscle fibers and then the small molecule inhibitor was sequentially added at concentrations of 2, 5, 10, and 20 μM. The muscle preparations were incubated for 20-30 minutes with NMD1226 or NMD653 before measurements were initiated. To evaluate the potency and target engagement of the small molecule inhibitors to the ClC-1 channel, the concentration-dependent reduction in the chloride driven G_m to 50 % (EC_{50}) was evaluated. ClC-1 inhibition is expected to cause a reduction in G_m since these channels are responsible for 80-90 % of total G_m during rest (2). Using this approach, NMD1226 had an EC_{50} of 3.7±0.2 μM (n=3) and NMD653 had an EC_{50} of 4.7±1.9 μM (N=7). Furthermore, the inhibitors caused a reduction in the rheobase current needed to elicit APs and triggered a higher number of APs at the largest current, both compatible with increased fiber excitability.

ClC-1 currents measured in CHO cells. An automated patch clamp system, Qpatch I system (Sophion Bioscience A/S, Ballerup, DK), was used to estimate the effect of the small molecules NMD1226 and NMD653 on ClC-1 currents in Chinese Hamster Ovary (CHO) cells stably expressing the human isoform of ClC-1 (gene: CLCN1), as a final step confirming NMD1226 and NMD653 as an inhibitor of ClC-1. Cells were obtained from ChanTest (Cat. No. CT6175, Charles River Laboratories, Cleveland, OH, USA). Detailed explanation of the method can be found elsewhere (16). In short, CHO cells and isotonic intracellular (IC) solution containing (mM): 80 CsF, 60 CsCl, 5/1 KOH/EGTA, 10 HEPES, 10 NaCl (pH=7.2, osmolality ~320 mOsm), and

extracellular (EC) solution containing (mM): 2 $CaCl_2$, 1 $MgCl_2$, 10 HEPES, 4 KCl, 145 NaCl, 10 Glucose (pH=7.4, osmolality ~320 mOsm) were prepared and loaded into the Qpatch system according to manufacturers' instructions. All IC and EC solutions contained 0.2% DMSO, which was the vehicle. The automated system hereafter established individual whole cell patches and cells were then clamped to a holding potential of -30mV. Subsequently, the voltage was increased to a pre-pulse of +60 mV for 100 ms where the maximum ClC-1 current was recorded when transiently shifted to -100 mV. This voltage protocol was repeated while the liquid exchange protocol was run. The liquid exchange protocol consisted of 4 steps: Cells were exposed to EC solution ("Saline control") (step 1), then cells were exposed to a new portion of EC solution in which NMD1226 or NMD653 was diluted to a concentration of 200 μM ("ClC-1 inhibitor") (step 2), hereafter NMD1226 or NMD653 was washed out using EC solution (step 3), and finally the whole cell patches were exposed to 9-anthracene-carboxylic acid (9-AC, established ClC-1 channel inhibitor) diluted in the EC solution to a concentration of 100 μM acting as a positive control (step 4). For data analysis, the maximum ClC-1 activation current amplitudes at +60 mV were extracted from the 4 steps in the liquid exchange protocol to determine whether NMD1226 and NMD653 indeed targeted the ClC-1 channel and to what level the molecule inhibited the channels compared to that of 9-AC. Successful inhibition of the ClC-1 channel would be shown through a decrease in the maximum ClC-1 current amplitude.

Force measurement in isolated intact nerve-muscle preparations. Ex vivo measurement of whole muscle force allowed for investigation of the ability of the small molecules to restore force after imposing partial neuromuscular blockade with tubocurarine. Experiments were conducted with whole soleus muscles dissected from 1-month-old rats. Chambers containing 20 mL NKR solution were pre-heated to 30 °C and subsequently all muscles were mounted on force transducers

(FORT 250, World Precision Instruments GmbH, Friedberg, DE) in the chambers. Then muscles were stretched to their optimal length and left to equilibrate for 30 minutes before the experiment was started and the muscles were stimulated at a range of frequencies. The acquisition program Signal (version 6.4, Cambridge Electronics Design Ltd, Cambridge, UK) was used to control stimulation, which was delivered by an isolated stimulator (Isostim 01D, NPI electronic GmbH, Tamm, DE), and to record data using an analogue-digital converter (Micro 1401 Cambridge Electronics Design Ltd, Cambridge, UK). For the duration of the experiment, muscles were stimulated both directly and via the nerve at 12 V. To achieve this, the stimulation pulse duration was changed between 0.02 ms for nerve stimulation and 0.2 ms for direct stimulation of muscle fibers. Muscles were stimulated every 10 minutes at 60 Hz for 2 s, either directly or via the nerve throughout the experiment. To test the effect of ClC-1 inhibition on force generation, force was first depressed through addition of 0.115 µM tubocurarine, an AChR antagonist, directly to the experimental chamber of individual muscles. After an incubation time of 90 minutes, a concentration of 50 µM of NMD1226 or NMD653 was added directly to the individual experimental chambers and allowed to recover to a steady elevated level(17).

Preclinical Stimulated Single Fiber EMG. Similar to the clinical studies, adult and aged rats underwent stimulated SFEMG assessment of jitter and NMJ blocking of the gastrocnemius muscle using a Sierra Summit clinical electrodiagnostic system (Cadwell, Kennewick, WA, USA) (10). Before initiating SFEMG measurements, rats were anaesthetized via inhalation of isoflurane delivered at 3-5% for induction and 2-3.5% for maintenance mixed with the ventilation gas. Both induction and maintenance of anesthesia occurred using a mask attached to the ventilation system (Hallowell MicroVent 1 Rodent Anesthesia Ventilator, Dre Veterinary, KY, USA) that was coupled to an isoflurane vaporizer (E-vet A/S, Haderslev, DK). The body temperature was

maintained around 37 °C by placing the rat on a heating pad. Eye lubricant was applied to avoid dryness and irritation of the eyes. Hair of the left hind leg was shaved, and the foot of the left hindlimb leg was secured with tape with the hip joint in an abducted position and the ankle in plantarflexion. Two stimulation electrodes (Monopolar EMG Needle Electrode 25mm x 27 g, Chalgren, London, UK) were then placed percutaneously at the sciatic nerve to electrically stimulate the gastrocnemius muscle through nerve stimulation. One ground electrode (subdermal needle electrode, Cadwell Kennewick, WA, USA) was inserted subcutaneously at the root of the tail. A highly selective SFEMG recording needle (25 x 0.30 mm, SEI EMG s.r.l., Cittadella, IT) was inserted into the gastrocnemius muscle to record single fiber APs elicited upon stimulation. The sciatic nerve was stimulated with a frequency of 10 Hz, pulse duration of 50 μs, and varying current intensity (mA) that were adapted to each rat. Criteria for the captured APs were a minimum amplitude of 200 μV and a rise time <500 μs together with an all-or-nothing response (18). One hundred (100) potentials were captured from each synapse, and around 8 synapses were assessed from each animal. Jitter (mean consecutive delay, MCD) was reported as an average per animal and blocking was reported as the percentage of synapses with blocking out of the total number of synapses for each animal.

Stimulated muscle force in anaesthetized animals. An experimental setup designed to enable measurements of stimulated muscle force from *triceps surae* muscle in anaesthetized and mechanically ventilated rats was used(19). Anesthesia was introduced with a 1:1 mix of Fentanyl (Hypnorm) and Midazolam (Dormicum 5mg/mL) (Hameln Pharma Plus GmbH, Hameln, DE) at dose volume 1 mL/kg subcutaneously. Hereafter, rats were intubated to enable mechanical ventilation, and a tube was inserted through the esophagus to the ventricle to allow P.O. dosing. Ventilation was controlled by a ventilator (Hallowell MicroVent 1 Rodent Anesthesia Ventilator,

Dre Veterinary, KY, USA) and anesthesia was maintained by mixing isoflurane (2-3%) in the ventilation gas through an isoflurane vaporizer (E-vet A/S, Haderslev, DK). The animals' core body temperature was monitored throughout the experiment and was maintained at 37.0-37.5 °C by a heating pad. Subsequently, a hind leg was set up for measurement of muscle force: The distal part of the *triceps surae* muscle was exposed and a string was tied firmly to the Achilles tendon after which the tendon was cut distally to the string. The string was then connected to a force transducer (FORT 1000, World Precision Instruments, FL, USA). Two stimulation electrodes (Monopolar EMG Needle Electrode 25mm x 27 g, Chalgren, London, UK) were placed percutaneously at the sciatic nerve to elicit contractile responses of the *triceps surae* muscle upon electrical stimulation. The position of the stimulation electrodes was adjusted until the best possible force response was obtained. Force generation elicited by stimulation was measured by the force transducer and recorded by the acquisition program Signal (version 6.4, Cambridge Electronics Design Ltd, Cambridge, UK) that was also used to control stimulation, delivered by an isolated stimulator (isostim 01D, NPI electronic GmbH, Tamm, DE) and recording of data via an analogue-digital converter (Micro 1401 Cambridge Electronics Design Ltd, Cambridge, UK).

After set-up, the sciatic nerve was stimulated with 10 pulses of 12 Hz (12-14 V) every 30 s (pulse duration of 0.1-0.4 ms). After 9 stimulations (5 minutes) the 12 Hz stimulation was substituted by 80 Hz stimulation train lasting 1 s. This cycle was repeated until the end of the experiment. Stimulated muscle force was allowed to stabilize (~2 h), if possible, whereafter 60 mg/kg NMD1226 was administered through the P.O. tube, as explained above. Subsequently the force was allowed to evolve. Muscle force data from 80 Hz stimulations was quantified and presented as Area Under the Curve (AUC) of the active force (g*s). While the administered compound was

selective for the ClC-1 channel, the dose requirements were high due to moderate affinity for ClC-1 channel.

7-day blinded dosing study. A 7-day blinded dosing study was performed to investigate the effect of ClC-1 inhibition in an aged rat model in an in vivo setting. Nine male 20-month-old Wistar rats were allocated into two treatment groups. The first group received 60 mg/kg NMD1226 at each dose (5 rats) and the second group received vehicle (sterile water) (4 rats). Treatments were administrated by oral gavage (P.O.) twice daily on study day 1-6 plus one time in the afternoon on study day 0 and one time in the morning on study day 7 (a total of 7 treatment days) (see Figure 4). Morning doses were administered around 07:30 AM and afternoon doses around 04:30 PM. The muscle strength of the 2 treatment groups was tested throughout the study using grip strength (GS) tests (see below). GS was performed at baseline prior to dose administration on study day 0, on study day 4 (between doses), on study day 7 (approximately 3 h after dosing), and on study day 8 (approximately 26 h after last dose, when treatments had been washed out) (see Figure 4). The treatment groups were blinded to the study investigator. Analysis of data was concluded before unblinding. Grip strength tests were used in the blinded study to measure voluntary muscle force as rats grasped a metal mesh connected to a force transducer while being gently pulled away. Valid attempts were averaged for analysis, with data acquired using Signal 6.4 software or Grip Strength Meter software (version 1.0.6.0, Columbus Instruments, OH, USA). In separate explorations, the half-life of the compound in rats upon P.O. dosing was found to be around 5 hours, and the time to reach maximum concentration was around 20 minutes (data not shown).

Grip strength test. GS tests were used to assess muscle strength in the blinded *in vivo* study. The GS test examined the voluntary muscle force that a rat was able to produce when grabbing on to a metal mesh attached to a force transducer (Grip Strength Meter, Columbus Instruments, Columbus,

OH, USA) while being pulled away. The rats were handled to allow all four paws to grab the mesh on the grip strength meter. Each rat was pulled 4 times by holding the base of the tail pulling in the opposite direction of its body orientation, i.e., towards the operator standing at the posterior direction of the rat and facing the apparatus. The rats were stimulated to do the active pulling, and the force recorded therefore reflected the grip strength of the rats and not the operator. However, these determinations can be challenging to perform, and this was one of the reasons for conducting this study with a blinded design. There was minimal pause between the repeated tests except for what the software needed for processing (few seconds). If a rat resisted to grip or pull, then one additional attempt was given. While performing a GS test, it was evaluated whether each pull was valid or invalid. Two training sessions were performed before starting the study for each animal. Average values of the valid attempts per test occasion were used for further data processing.

Data acquisition. Digital data was sampled using interfaces from Cambridge Electronic Design using Signal 6.4 software (CED, Cambridge, UK), Cadwell Sierra Summit EMG unit (Cadwell Industries, Kennewick, WA, USA) or software for the Grip Strength Meter version 1.0.6.0 (Columbus Instruments, Columbus, OH, USA).

Acute ClC-1 inhibition in aged mice: Compound Muscle Action Potential, Repetitive Nerve Stimulation, and stimulated single fiber electromyography. Aged C57BL/6J mice [n=11 (6 females, 5 males), mean 27.9 month, range 27-29 month], or vehicle [n=11 (4 females, 7 males), mean 28.3 months, range age 27-29] were randomly assigned to receive a single dose of the ClC-1 inhibitor NMD653 (20 mg/kg, intraperitoneal) or vehicle. All procedures were approved by the local Institutional Animal Care and Use Committee and conformed to NIH guidelines for the care and use of laboratory animals. Mice were anesthetized with isoflurane (3–5% for induction, 1.5–

2.5% for maintenance) and placed on a thermostatically controlled platform to maintain core temperature at 36.5–37.5°C.

For CMAP and RNS recordings, bipolar stimulating electrodes were positioned transcutaneously over the sciatic nerve at the mid-thigh, and recording electrodes were inserted into the gastrocnemius muscle, as described for rat experiments. Supramaximal stimuli were delivered using a Sierra Summit clinical electrodiagnostic system (Cadwell, Kennewick, WA, USA). Baseline CMAPs and RNS trains were acquired prior to dosing. RNS was performed using trains of 10 supramaximal stimuli at 10–50 Hz. CMAP amplitude was measured peak-to-peak for each response in the train, and percent decrement was calculated as previously described. Post-dose RNS measurements were repeated within 120 minutes to capture the peak pharmacodynamic effect of NMD653on neuromuscular transmission. Stimulated single-fiber EMG (SFEMG) was performed using a highly selective SFEMG needle electrode (25 × 0.30 mm, Natus neurology Inc, WI, USA) inserted into the gastrocnemius muscle, following the preclinical SFEMG protocol similar to that used in rats. The sciatic nerve was stimulated at 10 Hz (pulse duration 50 µs, current adjusted to achieve supramaximal activation) while the position of the recording electrode was adjusted to isolate single muscle fiber APs. Acceptable single-fiber APs met standard criteria of all-or-none response, rise time <500 µs, and amplitude ≥200 µV. For each mouse, 100 consecutive stimuli were recorded from each synapse, and jitter was quantified as mean consecutive difference (MCD). NMJ blocking was defined as the percentage of stimuli failing to elicit a muscle fiber AP. Jitter and blocking values were averaged across synapses to yield a single value per animal. All electrophysiologic analyses were performed by an investigator blinded to treatment group. Group comparisons for CMAP decrement and SFEMG jitter were made using unpaired t-tests with

Welch's correction, as described in the main text. Outliers were assessed a priori using the ROUT method (Q = 10%); one outlier was removed from the SFEMG jitter dataset prior to final analysis.

Statistics

Statistical analyses were prespecified and performed using SPSS Statistics version 27 (IBM, Chicago, IL) and GraphPad Prism (GraphPad Software, San Diego, CA, USA). Data distributions were assessed and parametric or non-parametric tests were selected accordingly. All statistical tests were two-tailed, and a pre-set alpha level of 0.05 was required for statistical significance. Data are presented as individual values or as mean ± SEM or SD, as indicated, and n-values represent the number of animals, muscles, experiments, or human participants analyzed.

Clinical Assessment of NMJ Transmission. Clinical analyses were conducted using SPSS version 27. Following confirmation of normality, group differences between young/middle-aged controls and older adults were analyzed using independent-samples t-tests. Associations between SFEMG parameters (jitter and blocking) and muscle strength normalized to quadriceps muscle volume were evaluated using quadratic regression, with correlation coefficients (*r*values) derived from the regression lines of best fit.

For SFEMG analyses, jitter was quantified as mean consecutive difference (MCD) and averaged at the subject level prior to statistical testing. Blocking was expressed as the percentage of synapses demonstrating transmission failure per subject.

Muscle Fiber Excitability, NMJ Excitability, and NaV1.4 Channel Staining. Data from muscle fiber electrophysiology, junctional versus extrajunctional excitability, and NaV1.4 immunohistochemical analyses were analyzed using GraphPad Prism. Unpaired t-tests were used

for normally distributed variables, and Mann–Whitney tests were used for non-parametric data. For analyses involving paired junctional and extra-junctional measurements within the same muscle fiber, paired comparisons were used as appropriate. For experiments involving multiple groups or compartments, analysis of variance (ANOVA) with appropriate post hoc corrections were applied.

Investigation of ClC-1 Inhibition to Enhance NMJ Transmission and Muscle Function. Data related to ClC-1 inhibition were analyzed using GraphPad Prism or SPSS version 27. Normality was assessed using Q–Q plots. Differences in SFEMG parameters between adult and old rats were analyzed using independent-samples t-tests with Welch's correction, as equal variances could not be assumed.

The acute effect of ClC-1 inhibition on nerve-stimulated muscle force was analyzed using a two-way repeated-measures ANOVA followed by Fisher's least significant difference (LSD) post hoc testing. The effect of 7 days of ClC-1 inhibitor dosing on voluntary grip strength was analyzed using a repeated-measures ANCOVA with group and time as factors and baseline strength as a covariate. Post hoc Fisher's LSD tests were used to interrogate significant main effects and interactions.

Force measurements obtained from isolated intact nerve–muscle preparations were analyzed using a one-way repeated-measures ANOVA followed by Fisher's LSD post hoc testing.

References

1. Riisager A, Duehmke R, Nielsen OB, Huang CL, and Pedersen TH. Determination of cable parameters in skeletal muscle fibres during repetitive firing of action potentials. *The Journal of physiology.* 2014;592(20):4417-29.
2. Jentsch TJ, and Pusch M. CLC Chloride Channels and Transporters: Structure, Function, Physiology, and Disease. *Physiol Rev.* 2018;98(3):1493-590.
3. Manini TM, Visser M, Won-Park S, Patel KV, Strotmeyer ES, Chen H, et al. Knee extension strength cutpoints for maintaining mobility. *J Am Geriatr Soc.* 2007;55(3):451-7.
4. Villafane JH, Valdes K, Vanti C, Pillastrini P, and Borboni A. Reliability of handgrip strength test in elderly subjects with unilateral thumb carpometacarpal osteoarthritis. *Hand (N Y).* 2015;10(2):205-9.
5. Guralnik JM, Simonsick EM, Ferrucci L, Glynn RJ, Berkman LF, Blazer DG, et al. A short physical performance battery assessing lower extremity function: association with self-reported disability and prediction of mortality and nursing home admission. *J Gerontol.* 1994;49(2):M85-94.
6. Wages NP, Simon JE, Clark LA, Amano S, Russ DW, Manini TM, et al. Relative contribution of muscle strength, lean mass, and lower extremity motor function in explaining between-person variance in mobility in older adults. *BMC Geriatr.* 2020;20(1):255.
7. Tavoian D, Ampomah K, Amano S, Law TD, and Clark BC. Changes in DXA-derived lean mass and MRI-derived cross-sectional area of the thigh are modestly associated. *Sci Rep.* 2019;9(1):10028.
8. Hangartner TN, Warner S, Braillon P, Jankowski L, and Shepherd J. The Official Positions of the International Society for Clinical Densitometry: acquisition of dual-energy X-ray absorptiometry body composition and considerations regarding analysis and repeatability of measures. *J Clin Densitom.* 2013;16(4):520-36.
9. Sanders DB, Arimura K, Cui L, Ertas M, Farrugia ME, Gilchrist J, et al. Guidelines for single fiber EMG. *Clin Neurophysiol.* 2019;130(8):1417-39.
10. Sanders DB, Arimura K, Cui L, Ertaş M, Farrugia ME, Gilchrist J, et al. Guidelines for single fiber EMG. *Clinical Neurophysiology.* 2019;130(8):1417-39.
11. Sleigh JN, Burgess RW, Gillingwater TH, and Cader MZ. Morphological analysis of neuromuscular junction development and degeneration in rodent lumbrical muscles. *J Neurosci Methods.* 2014;227:159-65.
12. Jones RA, Reich CD, Dissanayake KN, Kristmundsdottir F, Findlater GS, Ribchester RR, et al. NMJ-morph reveals principal components of synaptic morphology influencing structure-function relationships at the neuromuscular junction. *Open Biol.* 2016;6(12).
13. Niven DJ, Berthiaume LR, Fick GH, and Laupland KB. Matched case-control studies: a review of reported statistical methodology. *Clin Epidemiol.* 2012;4:99-110.
14. Proudfoot JA, Lin T, Wang B, and Tu XM. Tests for paired count outcomes. *Gen Psychiatr.* 2018;31(1):e100004.

15. Pedersen TH, de Paoli F, and Nielsen OB. Increased excitability of acidified skeletal muscle: role of chloride conductance. *J Gen Physiol.* 2005;125(2):237-46.
16. Accardi A, and Pusch M. Fast and slow gating relaxations in the muscle chloride channel CLC-1. *J Gen Physiol.* 2000;116(3):433-44.
17. Pedersen TH, Macdonald WA, Broch-Lips M, Halldorsdottir O, and Baekgaard Nielsen O. Chloride channel inhibition improves neuromuscular function under conditions mimicking neuromuscular disorders. *Acta Physiol (Oxf).* 2021;233(2):e13690.
18. Padilla CJ, Harrigan ME, Harris H, Schwab JM, Rutkove SB, Rich MM, et al. Profiling age-related muscle weakness and wasting: neuromuscular junction transmission as a driver of age-related physical decline. *Geroscience.* 2021;43(3):1265-81.
19. Winther JB, Morgen JJ, Skov M, Broch-Lips MG, Nielsen OB, Overgaard K, et al. Role of recovery of acetylcholine release in compromised neuromuscular junction function. *Neuromuscul Disord.* 2024;36:48-59.
